# Socioenvironmental Adversity and Adolescent Psychotic Experiences: Exploring Potential Mechanisms in a UK Longitudinal Cohort

**DOI:** 10.1093/schbul/sbad017

**Published:** 2023-03-19

**Authors:** Joanne B Newbury, Louise Arseneault, Terrie E Moffitt, Candice L Odgers, Laura D Howe, Ioannis Bakolis, Aaron Reuben, Andrea Danese, Karen Sugden, Benjamin Williams, Line J H Rasmussen, Antonella Trotta, Antony P Ambler, Helen L Fisher

**Affiliations:** King’s College London, Social, Genetic and Developmental Psychiatry Centre, Institute of Psychiatry, Psychology and Neuroscience, London, UK; Population Health Sciences, Bristol Medical School, University of Bristol, Bristol, UK; King’s College London, Social, Genetic and Developmental Psychiatry Centre, Institute of Psychiatry, Psychology and Neuroscience, London, UK; King’s College London, Social, Genetic and Developmental Psychiatry Centre, Institute of Psychiatry, Psychology and Neuroscience, London, UK; Department of Psychology and Neuroscience, Duke University, Durham, NC, USA; Department of Psychiatry and Behavioral Sciences, Duke University, Durham, NC, USA; Centre for Genomic and Computational Biology, Duke University, Durham, NC, USA; Social Science Research Institute, Duke University, Durham, NC, USA; Department of Psychological Science, School of Social Ecology, University of California, Irvine, Irvine, CA, USA; Population Health Sciences, Bristol Medical School, University of Bristol, Bristol, UK; King’s College London, Centre for Implementation Science, Department of Health Service and Population Research, Institute of Psychiatry, Psychology and Neuroscience, London, UK; King’s College London, Department of Biostatistics and Health Informatics, Institute of Psychiatry, Psychology and Neuroscience, London, UK; Department of Psychology and Neuroscience, Duke University, Durham, NC, USA; King’s College London, Social, Genetic and Developmental Psychiatry Centre, Institute of Psychiatry, Psychology and Neuroscience, London, UK; King’s College London, Department of Child and Adolescent Psychiatry, Institute of Psychiatry, Psychology and Neuroscience, London, UK; National and Specialist CAMHS Clinic for Trauma, Anxiety, and Depression, South London and Maudsley NHS Foundation Trust, London, UK; Department of Psychology and Neuroscience, Duke University, Durham, NC, USA; Department of Psychology and Neuroscience, Duke University, Durham, NC, USA; Department of Psychology and Neuroscience, Duke University, Durham, NC, USA; Department of Clinical Research, Copenhagen University Hospital Amager and Hvidovre, Hvidovre, Denmark; King’s College London, Social, Genetic and Developmental Psychiatry Centre, Institute of Psychiatry, Psychology and Neuroscience, London, UK; School of Health and Social Care, University of Essex, Colchester, UK; King’s College London, Social, Genetic and Developmental Psychiatry Centre, Institute of Psychiatry, Psychology and Neuroscience, London, UK; King’s College London, Social, Genetic and Developmental Psychiatry Centre, Institute of Psychiatry, Psychology and Neuroscience, London, UK; ESRC Centre for Society and Mental Health, King’s College London, London, UK

**Keywords:** disadvantage, intelligence, mediation, neighborhood, psychosis, urban

## Abstract

**Background and Hypothesis:**

Children exposed to socioenvironmental adversities (eg, urbanicity, pollution, neighborhood deprivation, crime, and family disadvantage) are more likely to subsequently develop subclinical psychotic experiences during adolescence (eg, hearing voices, paranoia). However, the pathways through which this occurs have not been previously investigated. We hypothesized that cognitive ability and inflammation would partly explain this association.

**Study Design:**

Data were utilized from the Environmental-Risk Longitudinal Twin Study, a cohort of 2232 children born in 1994–1995 in England and Wales and followed to age 18. Socioenvironmental adversities were measured from birth to age 10 and classified into physical risk (defined by high urbanicity and air pollution) and socioeconomic risk (defined by high neighborhood deprivation, neighborhood disorder, and family disadvantage). Cognitive abilities (overall, crystallized, fluid, and working memory) were assessed at age 12; and inflammatory markers (C-reactive protein, interleukin-6, soluble urokinase plasminogen activator receptor) were measured at age 18 from blood samples. Participants were interviewed at age 18 regarding psychotic experiences.

**Study Results:**

Higher physical risk and socioeconomic risk were associated with increased odds of psychotic experiences in adolescence. The largest mediation pathways were from socioeconomic risk via overall cognitive ability and crystallized ability, which accounted for ~11% and ~19% of the association with psychotic experiences, respectively. No statistically significant pathways were found via inflammatory markers in exploratory (partially cross-sectional) analyses.

**Conclusions:**

Cognitive ability, especially crystallized ability, may partly explain the association between childhood socioenvironmental adversity and adolescent psychotic experiences. Interventions to support cognitive development among children living in disadvantaged settings could buffer them against developing subclinical psychotic phenomena.

## Introduction

Psychotic disorders such as schizophrenia have a lifetime prevalence of around 3%^1^ and place a large burden on the individuals affected and society more broadly.^2^ Subclinical psychotic experiences (eg, hearing voices and paranoia) are considered to lie on a continuum with psychotic disorders and affect a greater proportion of the general population.^3^ These experiences are especially prevalent earlier in development, affecting up to a third of children and adolescents.^4–6^ As they are common and associate familially^7^ and longitudinally^8,9^ with psychotic disorders (with approximately a 7- to 16-fold increased risk of schizophreniform disorders in adulthood^8,9^), they provide an important and useful framework for investigating early-life risk factors for clinical psychosis. They also associate with a wide range of other current and subsequent mental health problems such as conduct disorder,^10^ self-harm and suicide attempts,^11^ depression,^12^ and PTSD,^8^ making them an important early marker of vulnerability for psychopathology more broadly. Moreover, early psychotic phenomena have also been associated with functional difficulties, risky behaviors, and poor quality of life in young adulthood.^11^ Therefore, understanding how subclinical psychotic experiences emerge is crucial to inform the development and testing of targeted preventive interventions in the general population.

Various socioenvironmental adversities appear to contribute to the development of psychotic disorders. These include urbanicity^13^ and a milieu of correlated exposures such as air pollution,^14^ neighborhood^15^ and family deprivation,^16^ and other neighborhood problems like crime and disorganization.^17,18^ Similar associations are seen for early psychotic experiences,^6,19–22^ indicating that the association between socioenvironmental adversities and psychosis has early-life origins (although causality has not been proven).

Given their pervasiveness, adversities such as high urbanicity, deprivation, and air pollution could be prime targets for interventions to potentially reduce the population burden of psychotic phenomena. However, little is known about the mechanism(s) linking these adversities to psychosis. Identifying pathways could open new avenues for preventive interventions. We are aware of only one such mechanistic study by Lewis et al.,^23^ which investigated a mediating role of IQ in the association of population density and neighborhood deprivation at birth with schizophrenia in Swedish men. To the best of our knowledge, there have been no such studies focusing on subclinical psychotic experiences thus highlighting an important gap in the existing literature.

In considering potential mechanisms, cognition, and inflammation are useful starting points because they provide insight into processes in the brain and body. Cognitive ability tests, such as the Wechsler Intelligence Scales, measure neurocognitive strengths, and weaknesses, thereby providing standardized estimates of knowledge acquisition and, arguably, noninvasive proxies of brain development and functioning.^24,25^ Additionally, inflammatory markers obtained from blood samples provide insight into systemic inflammation, high levels of which may signify chronic stress and underlying disease processes^26^ and can adversely affect multiple organs including the brain.^27^

**Fig. 1.**
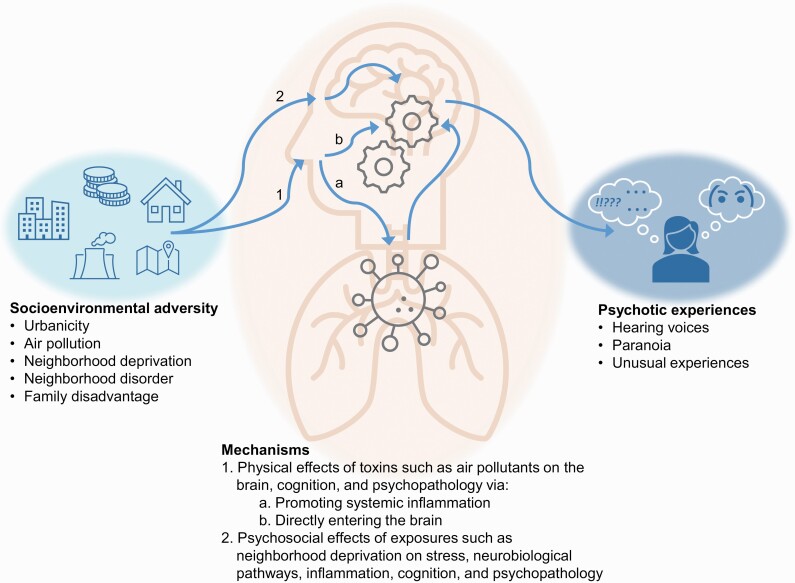
Diagram illustrating hypothesized pathways linking socioenvironmental adversities to psychotic experiences.

Figure 1 illustrates hypothesized pathways that could lead from socioenvironmental adversities, via cognitive ability and inflammation, to psychotic experiences. Briefly, physical toxins enriched in the urban environment, such as air pollutants, could promote oxidative stress and damage brain tissue by directly entering the brain.^28–30^ Exposures such as neighborhood deprivation and neighborhood disorder could also promote psychological stress and dysregulate neurobiological pathways linked to psychosis such as the hypothalamic–pituitary–adrenal axis^28^ and dopaminergic system.^31^ These processes could each have downstream effects on cognition and inflammation,^32–34^ both of which, based on longitudinal^35–38^ and mendelian randomization^39–42^ evidence, appear to have causal roles in psychosis.

It is likely that infants and children are most vulnerable to socioenvironmental adversities because their brains and immune systems are still developing.^43^ Therefore, in a longitudinal birth cohort followed to early adulthood, we conducted the first study to examine the roles of cognitive abilities and inflammation in the associations of socioenvironmental adversities in childhood with psychotic experiences in adolescence. We examined overall cognitive ability (IQ), and 3 subdomains (crystallized ability, fluid ability, and working memory) to explore potential specific neurocognitive pathways. Likewise, we examined 3 specific inflammatory biomarkers (C-reactive protein [CRP], interleukin-6 [IL-6], and soluble urokinase plasminogen activator receptor [suPAR]). Socioenvironmental adversities included urbanicity, air pollution, neighborhood deprivation, neighborhood disorder, and family disadvantage, and were selected *a* priori based on previous evidence in this cohort.^6,19–22^ However, analyses were guided by the exposome literature, which highlights the interdependent nature of associations between environmental exposures and mental disorders,^44^ and we, therefore, applied exploratory factor analysis to these socioenvironmental adversities. We hypothesized that cognitive ability and inflammation would partly mediate the association between socioenvironmental adversities and psychotic experiences.

## Methods

### Study Cohort

Participants were members of the Environmental Risk (E-Risk) Longitudinal Twin Study, which tracks the development of a nationally representative birth cohort of 2232 twin children born in 1994–1995 across England and Wales and initially assessed at age 5. Follow-up home visits were conducted when participants were aged 7, 10, 12, and 18 years (participation rates were 98%, 96%, 96%, and 93%, respectively). At 18 years of age, the E-Risk sample included 2066 participants, comprising 56.2% monozygotic twin pairs and 47.5% males. There were no differences between those who did and did not take part at age 18 in terms of age-5 socioeconomic status (SES) (*P* = .65), age-5 IQ scores (*P* = .33), or age-5 internalizing or externalizing behavioral problems (*P* = .69 and *P* = .68, respectively). E-Risk families are representative of UK households across the spectrum of neighborhood socioeconomic conditions (supplementary figure 1). The Joint South London and Maudsley and the Institute of Psychiatry Research Ethics Committee approved each phase of the study. Parents gave written informed consent, and participants gave written assent at ages 5–12 and written informed consent at age 18. Further details are reported elsewhere,^45^ and in supplementary material. Table 1 displays characteristics of the age-18 cohort.

### Measures

Socioenvironmental variables are described below, with more detail in supplementary material.

#### Urbanicity.

This was derived from classifications from 2011 census data,^46^ linked to home postcodes at ages 5, 7, and 10, and averaged across ages 5–10. A 3-level urbanicity variable was used representing rural (19.8%, *N* = 405), intermediate (47.8%, *N* = 980), and urban settings (32.4%, *N* = 665).

#### Air Pollution.

This exposure was measured using a coupled regional chemical transport model and street-scale dispersion model.^47,48^ Performance statistics are shown in supplementary table 1. Based on our previous findings,^6^ the present study focuses on nitrogen dioxide (NO_2_). Annualized estimates of ambient NO_2_ were linked to home postcodes at the age of 10 (*M* = 25.9μg/m^3^, *SD* = 10.2).

#### Neighborhood Deprivation.

This was based on a geodemographic discriminator that used over 400 census variables for Great Britain (CACI Information Services; http://www.caci.co.uk/).^49–51^ Classifications were linked to home postcodes at ages 5, 7, and 10, and then averaged across ages 5–10. Classifications ranged from 1 = “Wealthy Achiever” (19.9%, *N* = 410) to 5 = “Hard Pressed” (25.9%, *N* = 532) neighborhoods.

#### Neighborhood Disorder.

This was measured at age 5 via interviews with the children’s mothers.^52,53^ Mothers were asked whether 13 problems affected their neighborhood (eg, noisy neighbors, vandalism, burglaries, etc.). Items (coded 0–2) were summed for each mother (*M* = 3.95, *SD* = 3.82).

#### Family Disadvantage.

This was measured at the age of 5 as a composite of household social class (1 = unemployed/unskilled, 0 = part skilled, skilled manual, skilled non-manual, managerial/technical, professional); total household income (1 = ≤ £10 000 per year, 0 = > £10 000 per year); benefits excluding sickness benefit (1 = one or more benefit, 0 = no benefits); housing tenure (1 = local authority rental, 0 = homeowner, or private rental); and household car/van access (1 = no car/van, 0 = car owner).^49^ Half of children (48.8%, *N* = 1009) had experienced no family disadvantage and 3.9% (*N* = 80) had experienced all 5 forms of family disadvantage.

#### Adolescent Psychotic Experiences.

At age 18, participants were privately interviewed about 13 psychotic experiences they may have had since the age of 12 (ie, between 13 and 18 years of age). Seven items referred to delusions and hallucinations,^7^ such as “have you ever thought you were being watched, followed, or spied on?” and “do you hear voices that others cannot?.” Six items referred to unusual experiences, drawing from prodromal psychosis instruments including the PRIME-screen and Structured Interview for Prodromal Syndromes,^54^ such as “I believe I have special abilities or powers beyond my natural talents.” All 13 items (each coded 0, 1, or 2) were summed to create a psychotic experiences scale (*M* = 1.19, *SD* = 2.58, range = 0–18). Since there were low numbers of adolescents with high psychotic experiences scores (eg, only 1.0% [*N* = 21] had a score of 13+), scores were placed into an ordinal scale of 0 to 3 (0, 1–2, 3–5, and 6 or more psychotic experiences) to tackle the skewed distribution while retaining more information than a binary score. The distribution of this ordinal scale is shown in table 1. Further information is provided in supplementary material.

**Table 1.** Sample Characteristics and Missing Data for the Cohort at Age 18 (*N* = 2066)

Sample characteristics	M/N	SD/%	Range	Missing N (%)
Psychotic experiences				
None	1440	69.80%	—	3 (0.15)
1–2	319	15.46%	—	
3–5	166	8.05%	—	
6 or more	138	6.69%	—	
Overall cognitive ability	100.15	15.00	46.52–147.45	56 (2.71)
Crystallized ability	9.26	3.21	1–19	56 (2.71)
Fluid ability	9.50	2.80	1–19	57 (2.76)
Working memory	10.65	3.34	1–19	57 (2.76)
ln(CRP)	−0.08	1.39	−4.42–3.16	636 (30.78)
ln(IL-6)	−0.03	0.64	−5.00–2.66	626 (30.30)
suPAR	3.23	0.93	1.15–7.30	622 (30.11)
Physical risk factor score	1.80	0.63	0.74–2.73	87 (4.21)
Socioeconomic risk factor score	1.66	0.60	0.69–2.88	87 (4.21)
Separate socioenvironmental adversities:				
Urbanicity (ages 5–10)				
Rural	405	19.76%	—	16 (0.77)
Intermediate	980	47.80%	—	
Urban	665	32.44%	—	
Air pollution* (age 10)	25.94	10.17	2.59–57.87	75 (3.63)
Neighborhood deprivation (ages 5–10)				
Wealthy achiever	414	20.12%	—	8 (0.39)
Urban prosperity	223	10.84%	—	
Comfortably off	535	26.00%	—	
Moderate means	376	18.27%	—	
Hard pressed	510	24.78%	—	
Neighborhood disorder (age 5)	3.95	3.82	0 – 22	6 (0.29)
Family disadvantage (ages 0–5)				
None	1011	48.94%	—	0
1	350	16.94%	—	
2	246	11.91%	—	
3	201	9.73%	—	
4	178	8.62%	—	
5 forms of disadvantage	80	3.87%	—	
Covariates:				
Sex				
Male	981	47.48%	—	0
Female	1085	52.52%	—	
Family psychiatric history	0.37	0.27	0–1	56 (2.71)
Parental education				
At least CSE grade 0–5	1799	87.08%	—	0
None	267	12.92%	—	
PRS for schizophrenia	−0.02	1.00	−3.75–3.53	203 (9.83)
PRS for educational attainment	0.01	1.00	−3.70–3.31	232 (11.23)
PRS for cognitive performance	0.01	1.00	−2.93–2.80	232 (11.23)
Body temperature at age 18	36.34	0.58	34–38.2	13 (0.63)

*Note*: CRP, C-reactive protein, CSE, certificate of secondary education (school-leaving qualification), IL-6, interleukin-6, ln, natural logarithm, M, mean, N, number, PRS, polygenic risk score, SD, standard deviation, suPAR, soluble urokinase plasminogen activator receptor, *air pollution was nitrogen dioxide.

#### Cognitive Abilities.

At the age of 12, cognitive abilities were assessed using a short form of the Wechsler Intelligence Scale for Children-IV.^55^ Crystallized ability was measured via the Information task, fluid ability via the Matrix Reasoning task, and working memory via the Digit Span task. Crystallized ability is thought to reflect knowledge gained over time through learning (eg, vocabulary).^56,57^ Fluid ability is thought to reflect innate abilities in abstract reasoning and problem-solving (eg, puzzles).^58^ Working memory is thought to reflect abilities in retaining information for goal-directed behaviors (eg, attention). Overall cognitive ability was derived from these subdomains according to the procedure described by Sattler and Dumont.^59^

#### Inflammatory Biomarkers.

Venous blood was collected from 1700 participants at age 18. Plasma samples were available for 1448 participants. Biomarkers included CRP, IL-6, and an emerging biomarker, suPAR.^60^ Full details about these biomarkers are available elsewhere^61^ and in supplementary material. CRP is a protein produced by the liver. IL-6 is a cytokine with largely pro-inflammatory effects. suPAR is a protein that is cleaved from immunologically active cells when inflammation is higher. Both CRP and IL-6 are involved in the acute-phase response but may also reflect chronic inflammation. In contrast, suPAR is thought to provide a more stable indication of historic/chronic immune system activation.^60^

#### Covariates.

**Fig. 2.**
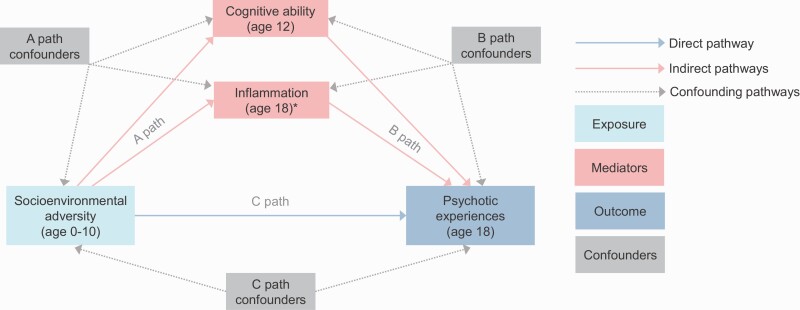
Directed acyclic graph illustrating tested mediation and confounding pathways between socioenvironmental adversities and psychotic experiences. Note: Socioenvironmental adversities were either the physical or socioeconomic risk factor scores (or the separate socioenvironmental adversities). Cognitive abilities included overall cognitive ability, crystallized cognitive ability, fluid cognitive ability, and working memory. Inflammation included C-reactive protein (CRP), interleukin-6 (IL-6), and soluble urokinase plasminogen activator receptor (suPAR). For mediation models, overall cognitive ability was included as a separate mediator. In contrast, specific cognitive abilities were included simultaneously to account for potentially overlapping pathways. Likewise, inflammatory markers were included simultaneously to account for potentially overlapping pathways. Directed acyclic graph was adapted from dagitty.net. *The analysis of inflammation as a potential mediator was exploratory because inflammatory markers were measured contemporaneously with psychotic experiences (at age 18). PRS, polygenic risk scores. (**A**) path confounders: For cognition—family psychiatric history, parental education, child’s polygenic risk score (PRS) for schizophrenia, child’s PRS for educational attainment, child’s PRS for cognitive performance. For inflammation—family psychiatric history, parental education, child’s PRS for schizophrenia. (**B**) path confounders: For cognition—same as A path confounders, plus biological sex at birth. For inflammation—same as A path confounders, plus biological sex at birth and body temperature at age 18. (**C**) path confounders: Family psychiatric history, parental education, child’s PRS for schizophrenia.

The selection of confounders was informed using a directed acyclic graph (figure 2). To account for potential confounding due to the selection of at-risk families into disadvantaged environments,^22^ we controlled for family psychiatric history, parental education, participants’ polygenic risk scores for schizophrenia, participants’ polygenic risk scores for educational attainment, and participants’ polygenic risk scores for cognitive performance. Additional covariates included biological sex and, for the inflammation analyses, body temperature at age 18. All covariates are described in supplementary material.

### Statistical Analyses.

The analysis plan was preregistered (https://sites.duke.edu/moffittcaspiprojects/files/2021/07/Newbury_2021a.pdf). Analyses were conducted in Stata v17.0. To recognize the exposome literature as well as to reduce data, we applied exploratory factor analysis to the socioenvironmental variables prior to the main analyses. Factor analysis was performed on the polychoric correlation matrix (principal factors with varimax rotation). This suggested a two-factor solution: correlations, variances, and factor loadings are shown in supplementary tables 2–4, and a scree plot is shown in supplementary figure 2. We extracted analysis variables directly from the two-factor solution, using the “predict” command in Stata. The first factor, conceptualized as “physical risk,” had the highest loadings from urbanicity and air pollution, as well as smaller loadings from the other three variables (*M* = 1.80, *SD* = 0.63, range = 0.74–2.73). The second factor, conceptualized as “socioeconomic risk,” had the highest loadings from neighborhood deprivation, neighborhood disorder, and family disadvantage, as well as smaller loadings from the other 2 variables (*M* = 1.66, *SD* = 0.60, range = 0.69–2.88). The correlation between the two factors was *r* = 0.08 (*P* < .001).

For the main analyses, we first conducted regression analysis to examine the associations of socioenvironmental adversities with psychotic experiences (ordinal logistic regression) and with cognitive ability and inflammation (linear regression). Second, ordinal logistic regression was used to examine the longitudinal association of cognitive ability with adolescent psychotic experiences and the cross-sectional association of inflammation with adolescent psychotic experiences. Third, generalized structural equation modeling (gsem) was used to partition the total effect of socioenvironmental adversities on psychotic experiences into the direct effect (not mediated via putative mediators) and the indirect (mediated) effects via cognitive abilities. Mediation pathways are shown in figure 2. Overall cognitive ability was analyzed as a separate mediator. Cognitive ability subdomains were analyzed as mediators simultaneously to account for potentially correlated pathways. We then conducted a supplementary mediation analysis of inflammation instead of cognitive ability, with inflammatory biomarkers analyzed simultaneously. This was considered exploratory because inflammation was measured contemporaneously to psychotic experiences at age 18 and therefore did not achieve the appropriate temporal sequencing usually required for mediation. Main analyses focused on the physical and socioeconomic risk factor scores. We conducted supplementary analyses for the separate socioenvironmental variables that comprised the factor scores. Finally, we calculated e-values^62^ where evidence of mediation was strongest. E-values indicate the strength of unmeasured confounding required to nullify associations and are recommended for observational research because unmeasured confounding is inevitable.^62^ All analyses (including mediation models) controlled for the non-independence of twin observations using the “cluster” command. This procedure is derived from the Huber-White variance estimator, and provides robust standard errors adjusted for within cluster (ie, within the family) correlated data.^63^ To handle potential issues arising from missing data, all analyses were conducted following multiple imputations by chained equations, described in supplementary material. We also conducted complete case analyses for main models.

## Results

### Sample Characteristics

Distributions for the main variables and covariates are described in table 1. At age 18, just under a third of adolescents (*N* = 626; 30.2%) reported having at least one psychotic experience after the age of 12.

### Are Socioenvironmental Adversities Associated With Psychotic Experiences?

Higher physical risk (adjusted odds ratio [adjOR] = 1.35, 95% CI = 1.13–1.61, *P* = .001) and socioeconomic risk (adjOR = 1.72, 95% CI = 1.42–2.08, *P* < .001) in childhood were associated with increased odds for psychotic experiences in adolescence.

### Are Socioenvironmental Adversities Associated With Cognitive Ability and Inflammation?

**Fig. 3.**
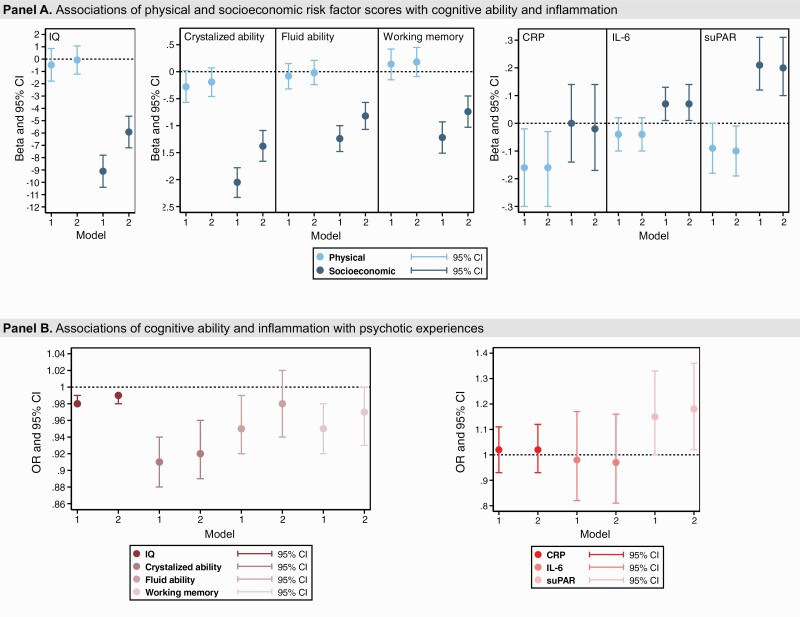
Associations of physical and socioeconomic risk factor scores with cognitive ability and inflammation (Panel A); and of cognitive ability and inflammation with psychotic experiences (Panel B). Note: CI, confidence interval, CRP, C-reactive protein, IL-6, interleukin-6, IQ, intelligence quotient (overall cognitive ability), OR, odds ratio, suPAR, soluble urokinase plasminogen activator receptor. Model 1-unadjusted. Model 2-adjusted (see Methods and figure 2 for model adjustments).

Higher socioeconomic risk up to the age of 10 was associated with lower overall cognitive ability, crystallized ability, fluid ability, and working memory at the age of 12, before and after covariate adjustment (supplementary table 5). For instance, each unit increase in the socioeconomic risk factor score was associated with a 5.92-IQ-points decrease in overall cognitive ability (adjusted beta [adjB] = −5.92, 95% CI = −7.20–−4.64). Effect sizes were smaller and CIs included the null for associations between physical risk and cognitive abilities (figure 3 [Panel A] and supplementary table 5).

Higher socioeconomic risk up to the age of 10 was also associated with higher IL-6 and suPAR levels at age 18. For instance, increases in the socioeconomic risk factor score were associated with 0.20ng/ml higher suPAR levels (adjB = 0.20, 95% CI = 0.10–0.31). Unexpectedly, the higher physical risk was associated with lower CRP and suPAR levels. For instance, increases in the physical risk factor score were associated with 0.09ng/ml lower suPAR levels (adjB = −0.09, 95% CI = −0.18–0.00) (figure 3 [Panel A] and supplementary table 5).

### Are Cognitive Ability and Inflammation Associated With Psychotic Experiences?

Higher scores for each of the cognitive abilities were associated with lower odds for adolescent psychotic experiences, though confidence intervals crossed the null for fluid ability following covariate adjustment (figure 3 [Panel B] and supplementary table 6). For instance, each unit increase in crystallized ability was associated with 9% lower odds for psychotic experiences (adjOR = 0.92, 95% CI = 0.89–0.96).

Associations between inflammation and psychotic experiences in this cohort have been reported previously,^64^ but we report associations again for the present analytic sample. Each unit increase in suPAR was associated with 18% greater odds for psychotic experiences (adjOR = 1.18, 95% CI = 1.02–1.36) (figure 3 [Panel B] and supplementary table 6). Neither CRP nor IL-6 was associated with psychotic experiences.

### Does Cognitive Ability Mediate the Association of Socioenvironmental Adversity With Psychotic Experiences?

**Fig. 4.**
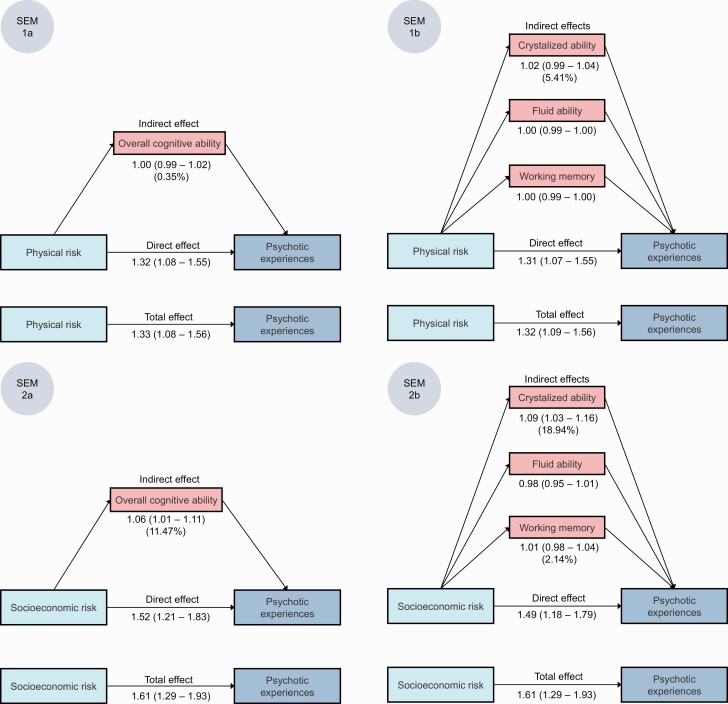
Mediation of the associations between socioenvironmental adversity and adolescent psychotic experiences, via cognitive ability. Note: SEM, structural equation model. Effect estimates are reported as OR (95% CI) and are fully adjusted. Model adjustments are described in the Methods and in figure 2. If mediation was detected, mediation percentages are shown in the figure underneath indirect effects. All analyses were conducted following multiple imputations and control for the non-independence of twin observations. Total effect: total association between physical/socioeconomic risk and psychotic experiences. Direct effect: part of the total effect that is not mediated (explained) by the putative mediator. Indirect effect: part of the total effect that is mediated (explained) by the putative mediator. Note that the indirect effects across the separate SEMs are not additive. Setup of structural equation models: 1a: exposure = physical risk, mediator = overall cognitive ability. 1b: exposure = physical risk, mediators = crystallized ability, fluid ability, working memory. 2a: exposure = socioeconomic risk, mediator = overall cognitive ability. 2b: exposure = socioeconomic risk, mediators = crystallized ability, fluid ability, working memory.

Figure 4 shows the adjusted total effects of the physical and socioeconomic risk factor scores on adolescent psychotic experiences, the direct effect (unmediated by cognitive abilities), the indirect effects mediated via cognitive abilities, as well as mediation percentages. Supplementary table 7 additionally shows unadjusted results.

Overall cognitive ability mediated ~11% (indirect adjOR = 1.06, 95% CI = 1.01–1.11) and crystallized ability ~19% (indirect adjOR = 1.09, 95% CI = 1.03–1.16) of the total effect of socioeconomic risk on psychotic experiences. In contrast, the only slight signal of mediation from physical risk was via crystallized ability, and this effect was small (~5%) and confidence intervals crossed the null. Furthermore, there was no evidence of mediation via fluid ability or working memory.

Notably, direct effects remained strong after considering mediation pathways, indicating that a large proportion of the association between socioenvironmental adversity and psychotic experiences was not explained by cognitive ability.

### Supplementary Analyses

We conducted an exploratory partially cross-sectional mediation analysis using inflammatory biomarkers. The only slight signal of mediation was from socioeconomic risk via suPAR, and this effect was small (~5%) and confidence intervals crossed the null (supplementary table 7). There was no evidence of mediation via CRP or IL-6.

In keeping with the main findings, all separate socioenvironmental adversities were associated with lower cognitive ability scores, particularly overall cognitive ability and crystallized ability (supplementary tables 8–12). Higher neighborhood deprivation, neighborhood disorder, and family disadvantage were also associated with higher suPAR levels. Unexpectedly (though in keeping with the main findings) urbanicity and air pollution were associated with lower CRP and suPAR levels (supplementary tables 8–12). However, there was evidence of mediation only via overall cognitive ability, which mediated the effects of deprivation, disorder, and disadvantage; and crystallized ability, which additionally mediated the effect of urbanicity (supplementary tables 13–22).

### E-values

E-values were calculated for the socioeconomic risk—crystallized ability–psychotic experience mediation model and are shown in supplementary table 23 alongside the associations of covariates with exposure, mediator, and outcome. E-values for the total (OR = 2.61, lower CI = 1.90) and direct effects (OR = 2.34, lower CI = 1.64) were relatively large, and usually close to or greater than the magnitude of associations of included covariates with exposure, mediator, and outcome. The e-value for the indirect effect was smaller (OR = 1.40, lower CI = 1.21), though still greater than the effect sizes for most covariates. This suggests that any unmeasured confounder(s) would require a stronger confounding influence than most of the included covariates to nullify associations.

Results from complete case analyses were similar to those from imputed data (supplementary tables 24–26).

## Discussion

This study assessed the mechanisms linking socioenvironmental adversities to psychotic experiences. Children exposed to more socioenvironmental adversity (both physical and socioeconomic) had greater odds of psychotic experiences in adolescence. Additionally, physical and socioeconomic risk were associated with several cognitive abilities and inflammatory markers; some of which, in turn, were associated with psychotic experiences. However, we found robust evidence only for overall cognitive ability and crystallized ability as mediators, which explained, respectively, ~11% and ~19% of the association between socioeconomic risk and psychotic experiences.

Our findings are partly consistent with those from Lewis et al.,^23^ who reported that IQ at age 18 explained 23% of the association between neighborhood deprivation at birth and schizophrenia in adulthood; though it did not mediate the association between urbanicity and schizophrenia. In our study, as well as mediating the socioeconomic risk-psychotic experience association, crystallized ability also explained a small proportion of the association of urbanicity with psychotic experiences, which could suggest a particular role of this subdomain of cognition.

A mediation pathway via cognitive ability, especially crystallized ability, is plausible and aligns with the neurodevelopmental model of psychosis, which describes the importance of early-life vulnerabilities and adversities for increasing neural abnormalities that later result in psychotic disorders.^65^ Though the nature of crystallized ability remains equivocal, it represents information stores acquired through learning and experience.^56,57,66^ Thus, of the domains we examined, crystallized ability is arguably subject to the most influence from socioenvironmental adversities. Notably, deficits in crystallized ability are associated with reduced cortical thickness,^67,68^ which is itself associated with psychosis.^69–71^

Our findings are consistent with a process whereby socioenvironmental adversities impact neurodevelopment during childhood, leading to reduced crystallized ability and an increased propensity for psychotic experiences in adolescence. However, a non-mutually exclusive psychosocial mechanism could also underlie these findings, whereby children growing up in disadvantaged environments face barriers to learning at school; and later, with reduced opportunities during the transition to adulthood, encounter more psychological stress which precipitates the emergence of psychotic experiences. For both suggested mechanisms, interventions to support cognitive development among children could help to weaken the effect of socioenvironmental adversity on psychotic experiences. Notably, this accords with research into cognitive remediation in psychosis which suggests that cognitive training improves functioning and reduces clinical transition among adolescents and young adults at ultra-high risk for psychosis.^72,73^

Unexpectedly, there was a dissociation in effects for inflammation. Socioeconomic risk was associated with higher IL-6 and suPAR levels, whereas, physical risk was associated with lower CRP and suPAR levels; a pattern reflected by the separate socioenvironmental variables. This finding brought to mind recent mendelian randomization evidence suggesting a negative causal relationship between CRP and schizophrenia, whereby high levels appeared protective.^41,42^ Together, these findings could suggest a complex interplay between socioenvironmental adversities, inflammation, and psychosis; with dysregulation, rather than increase, being the hallmark of associations. Further mendelian randomization research could shed light on these dissociated patterns.

### Strengths and Limitations

Our study had several strengths, including a longitudinal design, use of comprehensive data on several socioenvironmental adversities across childhood, and exploration of subcomponents of cognitive ability and inflammation. Moreover, E-Risk families are representative of the UK population in terms of urbanicity^46^ and socioeconomics.^51^

We also highlight some limitations. Inflammation was measured at age 18, meaning that we could not achieve the appropriate temporal sequence to properly explore inflammation as a mediator, and these results (null or weak mediation) are thus interpreted with caution. A powerful approach would be to measure biomarkers at multiple time points across development to obtain a longer-term picture of systemic inflammation. However, we used the novel biomarker suPAR, which may better capture the longer-term inflammatory consequences^60^ of socioenvironmental adversity, as shown previously for other types of childhood adversity.^74^ It was interesting that there was (weak) evidence of mediation only for this biomarker. Additionally, mediation models assume there is no unmeasured confounding. Though we identified potential confounders via a directed acyclic graph, residual confounding is inevitable. However, the reasonably large e-values increased our confidence in the findings. Additionally, we examined only two potential mechanisms, and the mediation pathway via the strongest mediator (crystallized ability) was still relatively modest, suggesting that a large proportion of the association between the socioenvironmental adversity and psychotic experiences is explained by different mechanisms. Future studies should explore other potential pathways between socioenvironmental adversity and psychotic experiences, such as childhood maltreatment and bullying; as well as processes upstream of cognitive abilities and inflammation, such as glucocorticoid levels. Furthermore, it is likely that some participants first had psychotic experiences after age 18, potentially in the context of schizophrenia, and were therefore missed by our study. Relatedly, it is unclear whether these findings would generalize to psychotic disorder, although they do align with Lewis et al.’s^23^ findings on schizophrenia. Finally, the causality of the association between socioenvironmental adversity and psychosis remains equivocal, with evidence that it could partly be driven by the selection of individuals with higher genetic risk into deprived^75^ and urban^76^ settings. However, we have previously shown in this cohort that these associations persist after controlling for numerous measures of genetic risk.^22^

## Conclusions

Our study builds on emerging evidence that cognitive ability occupies a pathway between socioenvironmental adversities and psychosis. We extend this evidence to early psychotic experiences and specific cognitive abilities. If replicated, these findings suggest that interventions to enhance cognitive development during childhood could increase resilience to psychotic experiences and potentially later psychopathology among children raised in disadvantaged circumstances.

## Supplementary Material

sbad017_suppl_Supplementary_Materials
