# Supplementary material for: Socioenvironmental Adversity and Adolescent Psychotic Experiences: Exploring Potential Mechanisms in a UK Longitudinal Cohort
Source: Schizophr Bull. 2023 Mar 19;49(4):1042–54. doi: 10.1093/schbul/sbad017 (PMC10318878; doi:10.1093/schbul/sbad017)
Supplement: sbad017_suppl_Supplementary_Materials [file sbad017_suppl_supplementary_materials.docx]

**Supplementary Online Content**

J. B. Newbury, L. Arseneault, T. E. Moffitt, C. L. Odgers, L. Howe, I. Bakolis, A. Reuben, A. Danese, K. Sugden, B. Williams, L. J. H. Rasmussen, A. Trotta, A. P. Ambler, and H. L. Fisher. Socioenvironmental adversity and adolescent psychotic experiences: exploring potential mechanisms in a UK longitudinal cohort

**Table of Contents for Supplementary Materials**

| **Supplementary Methods** | 4 |
| --- | --- |
| **Supplementary Figure 1.** Distribution of E-Risk participants at age 18 across Index of Multiple Deprivation deciles | 10 |
| **Supplementary Table 1.** Performance statistics of CMAQ-Urban for 2004 | 11 |
| **Supplementary Table 2.** Polychoric correlation matrix between socioenvironmental variables | 12 |
| **Supplementary Table 3.** Variance explained by two-factor solution | 13 |
| **Supplementary Table 4.** Rotated factor loadings and uniqueness | 14 |
| **Supplementary Figure 2.** Scree plot from factor analysis | 15 |
| **Supplementary Table 5.** Association of socioenvironmental adversity with cognitive ability and inflammation | 16 |
| **Supplementary Table 6.** Association of cognitive ability and inflammation with adolescent psychotic experiences | 17 |
| **Supplementary Table 7.** Mediation of the association between socioenvironmental adversity and adolescent psychotic experiences, via cognitive ability and inflammation | 18 |
| **Supplementary Table 8.** Association of urbanicity with cognitive ability and inflammation | 20 |
| **Supplementary Table 9.** Association of air pollution with cognitive ability and inflammation | 21 |
| **Supplementary Table 10.** Association of neighborhood deprivation with cognitive ability and inflammation | 22 |
| **Supplementary Table 11.** Association of neighborhood disorder with cognitive ability and inflammation | 23 |
| **Supplementary Table 12.** Association of family disadvantage with cognitive ability and inflammation | 24 |
| **Supplementary Table 13.** Association between urbanicity and adolescent psychotic experiences, mediated via cognitive ability | 25 |
| **Supplementary Table 14.** Association between air pollution and adolescent psychotic experiences, mediated via cognitive ability | 26 |
| **Supplementary Table 15.** Association between neighborhood deprivation and adolescent psychotic experiences, mediated via cognitive ability | 27 |
| **Supplementary Table 16.** Association between neighborhood disorder and adolescent psychotic experiences, mediated via cognitive ability | 28 |
| **Supplementary Table 17.** Association between family disadvantage and adolescent psychotic experiences, mediated via cognitive ability | 29 |
| **Supplementary Table 18.** Association between urbanicity and adolescent psychotic experiences, mediated via inflammation | 30 |
| **Supplementary Table 19.** Association between air pollution and adolescent psychotic experiences, mediated via inflammation | 31 |
| **Supplementary Table 20.** Association between neighborhood deprivation and adolescent psychotic experiences, mediated via inflammation | 32 |
| **Supplementary Table 21.** Association between neighborhood disorder and adolescent psychotic experiences, mediated via inflammation | 33 |
| **Supplementary Table 22.** Association between family disadvantage and adolescent psychotic experiences, mediated via inflammation | 34 |
| **Supplementary Table 23.** Comparison between e-values and covariate associations for the socioeconomic risk-crystalized ability-psychotic experiences mediation model | 35 |
| **Supplementary Table 24.** Complete case analysis: Association of socioenvironmental adversity with cognitive ability and inflammation | 36 |
| **Supplementary Table 25.** Complete case analysis: Association of cognitive ability and inflammation with adolescent psychotic experiences | 37 |
| **Supplementary Table 26.** Complete case analysis: Association between socioenvironmental adversity and adolescent psychotic experiences, mediated via cognitive ability and inflammation | 38 |
| **References from Supplementary Materials** | 40 |

**Supplementary Methods**

*Sample*

Participants were members of the Environmental Risk (E-Risk) Longitudinal Twin Study, which investigates how genetic and environmental factors shape children’s development. The sampling frame from which the E-Risk families were drawn was two consecutive birth cohorts (1994 and 1995) in a birth register of twins born in England and Wales.^1^ Of the 15,906 twin pairs born in these two years, 71% joined the register. The E-Risk Study probability sample was drawn using a high-risk stratification strategy. High-risk families were those in which the mother had her first birth when she was 20 years of age or younger. This sampling frame was used (1) to replace high risk families who were selectively lost to the register via non-response and (2) to ensure sufficient base rates of environmental risk factors. Age at first childbearing was used as the risk-stratification variable because it was present for virtually all families in the register, it is relatively free of measurement error, and early childbearing is associated with a host of other difficulties and is a known risk factor for children’s problem behaviors.^2,3^ The high-risk sampling strategy resulted in a final sample in which one-third of Study mothers constitute a 160% oversample of mothers who were at high risk based on their young age at first birth (13–20 years), while the other two-thirds of Study mothers accurately represent all mothers in the general population (13–48 years) in England and Wales in 1994–95 (estimates derived from the General Household Survey).^4^

The Study sought a sample size of 1,100 families to allow for attrition in future years of the longitudinal study while retaining statistical power. An initial list of families who had same-sex twins was drawn from the register to target for home-visits, with a 10% oversample to allow for nonparticipation. Of the 1,203 families from the initial list who were eligible for inclusion, 1,116 (93%) participated in home-visit assessments when the twins were age 5 years, forming the base sample for the study (2,232 children): 4% of families refused, and 3% were lost to tracing or could not be reached after many attempts. With parent’s permission, questionnaires were posted to the children’s teachers, and teachers returned questionnaires for 94% of cohort children. Zygosity was determined using a standard zygosity questionnaire, which has been shown to have 95% accuracy.^5^ Ambiguous cases were zygosity-typed using DNA. Subsequently, all participants who provided a DNA sample at any point across the study phases (97%) have been genotyped and had their zygosity checked. The sample includes 56% monozygotic (MZ) and 44% dizygotic (DZ) twin pairs. Sex is evenly distributed within zygosity (49% male). All families are English speaking, and the majority (93.7%) are White.

Attrition has been minimal, and data has been successfully collected from 98% (at age 7 years), 96% (at age 10 years), 96% (at age 12 years), and most recently in 2012–2014, 93% of the original sample (at age 18 years). Home-visits at ages 5, 7, 10, and 12 years included face-to-face assessments with participants as well as their mother (or primary caregiver); the home-visit at age 18 included interviews only with the participants, and questionnaires completed by co-informants (caregivers and other family members). Each twin participant was assessed by a different interviewer. Most participants (71.4%; N=1475) lived at the same address between ages 12 and 18. In addition, adolescents who did move house tended to move to similar neighborhoods: 87.0% of movers who lived in urban/intermediate neighborhoods at age 12 also lived in urban/intermediate neighborhoods at age 18.

The Joint South London and Maudsley and the Institute of Psychiatry Research Ethics Committee approved each phase of the study. Parents gave informed consent at ages 5–12. Participants gave assent at ages 5–12 and informed consent at age 18.

*Socioenvironmental risks.* Some variables were categorical and some were continuous. All continuous variables were placed into tertiles to allow consistent analyses and comparison between variables. In addition, categorical variables with more than 3 categories were collapsed into 3 categories, again to allow consistent analyses between the variables.

*Urbanicity* was derived from the Office for National Statistics’ (ONS) Rural-Urban Definition for Small Area Geographies (RUC2011) classifications.^6^ The ONS classifications utilized 2011 census data and were designed for application to small geostatistical units (e.g. Output Areas). Detailed information on how the ONS created the RUC2011 classifications of urbanicity is available on the ONS webpages (<https://www.gov.uk/government/uploads/system/uploads/attachment_data/file/239477/RUC11methodologypaperaug_28_Aug.pdf>). RUC2011 was created by laying a grid of hectare cells (100m^2^) over England and Wales. Postcode addresses were assigned to cells, and residential densities were then calculated for increasing radii around each cell, providing each residential property with a density profile. This was combined with Output Area and contextual data such as settlement size and population sparsity, allowing each settlement to be assigned to one of ten urbanicity categories (Rural categories: sparse/non-sparse hamlets and isolated dwellings, sparse/non-sparse villages, sparse/non-sparse rural town and fringe; Urban categories: sparse/non-sparse city and town, and minor/major conurbations [conurbations are densely populated, large urban regions resulting from the expansion and coalescence of adjacent cities and towns]). ONS urbanicity scores were then assigned to every E-Risk family via the family’s postcode when children were aged 5, 7 and 10. Given the low numbers within some rural categories, urbanicity was collapsed into three levels (1: “rural” = all rural categories; 2: “intermediate” = urban cities and towns; and 3: “urban” = minor/major conurbations). Urbanicity score was averaged across ages 5-10 and then rounded to the nearest integer (for the age 18 sample: rural=19.8%, N=405; intermediate=47.8%, N=980; urban=32.4%, N=665). E-Risk participants are nationally-representative in terms of ONS urbanicity classifications; for instance, the nationwide distribution of rural, intermediate, and urban residents in the UK is 18.9%, 45.0%, and 36.1%, respectively.

*Air pollution* exposure estimates were modelled and linked to the latitude-longitude coordinates of participants’ home addresses at age 10. Pollution estimates were modelled using CMAQ-urban, which is a coupled regional chemical transport model and street-scale dispersion model. CMAQ-urban uses a new generation of road traffic emissions inventory in the UK to model air quality down to individual streets, providing hourly estimates of pollutants at 20x20 metre grid points throughout the UK (i.e., address-level). Full details on the creation and validation of this model have been described previously.^7,8^ The pollution estimates achieved good model performance against ground-based measurements (see Supplementary Table 1). The present study focusses on nitrogen dioxide (NO_2_), a regulated gaseous pollutant, which has previously been shown to be associated with adolescent psychotic experiences in this sample.^9^ Participants’ exposure to NO_2_ was estimated by averaging hourly concentrations across the year (age 10/2004 exposure for the age 18 sample: M=25.94µg/m^3­­­^, SD=10.17, range=2.59-57.87). The model achieves 20x20 meter resolution. For this study, annualized NO_2_ exposure was categorized into tertiles (1=least polluted; 3=most polluted).

*Neighborhood deprivation* was constructed using A Classification of Residential Neighborhoods (ACORN), a geodemographic discriminator developed by CACI Information Services (CACI Information Services; http://www.caci.co.uk/). Detailed information about ACORN’s classification of neighborhood-level socioeconomic-status (SES) has been provided previously.^10-12^ Briefly, CACI utilized over 400 census variables for Great Britain (e.g., educational qualifications, unemployment, housing tenure) and CACI’s consumer lifestyle database. Classifications ranged from “Wealthy Achiever” (coded 1) to “Urban Prosperity” (coded 2), to “Comfortably Off” (coded 3), to “Moderate Means” (coded 4), to “Hard Pressed” (coded 5) neighborhoods. Each family in our sample was matched to the ACORN code for its neighborhood via postcodes at ages 5, 7, and 10.^10^ We averaged scores across ages 5-10 and rounded to the nearest integer and grouped ACORN codes 1 together with 2, and 3 together with 4 (for the age 18 sample: 1+2=29.8%, N=612; 3+4=44.41%, N=894; 5=25.85%, N=532).

*Neighborhood disorder* was measured at age 5 via in-home interviews with the children’s mothers.^13,14^ Mothers were asked whether thirteen problems affected their neighborhood, including noisy neighbors, arguments or loud parties; teenagers hanging around; drunk or homeless people; homes and gardens in bad condition; vandalism, graffiti or deliberate damage to property; homes broken into; cars broken into or stolen; animals running loose; bad smells; inadequate public transport; not enough local shops; quantity of available schools; and noisy traffic. Items (each coded 0–2) were summed for each mother (for the age 18 sample: M=3.95, SD=3.82). Scores were grouped into tertiles for analysis at the 33rd and 66th centile (1=lowest neighborhood disorder; 3=highest neighborhood disorder).

*Family disadvantage* was measured at age 5 as a composite of 5 binary variables: household social class (1=unemployed/unskilled, 0=part skilled, skilled manual, skilled non-manual, managerial/technical, professional); total household income (1=<£10,000 per year, 0= >£10,000 per year); benefits excluding sickness benefit (1=one or more benefit, 0=no benefits); housing tenure (1=local authority rental, 0=home owner or private rental); and household car/van access (1=no car/van, 0=car owner).^13^ The resulting variable ranged from 0 (no family disadvantage) to 5 (5 forms of family disadvantage). To allow comparison across socioenvironmental variables we grouped scores 1 and 2, and 3 or more for analysis (for the age 18 sample: 0=48.84%, N=1,009; 1 or 2=29.05%, N=598; 3 or more=22.22%, N=459).

*Adolescent psychotic experiences.* To measure adolescent psychotic experiences, E-Risk families were visited by mental health trainees or professionals when children were aged 18. Interviewers had no prior knowledge about the participant. Each participant was privately interviewed about thirteen psychotic experiences they may have experienced since the age of 12 (i.e., occurring between 13-18 years of age), including seven items pertaining to hallucinations and delusions, such as: Have other people ever read your thoughts? Have you ever believed that you were being sent special messages through the television or radio, or that a programme has been arranged just for you alone? Have you ever thought you were being followed or spied on? Have you ever heard voices that other people cannot hear? Have you ever felt like you were under the control of some special power? Have you ever known what another person was thinking, like you could read their mind? Have you ever seen something or someone that other people could not see?; as well as six items about unusual feelings and thoughts such as: I have become more sensitive to lights or sounds, I feel as though I can’t trust anyone, I worry that my food may be poisoned, People or places I know seem different, I believe I have special abilities or powers beyond my natural talents, and My thinking is unusual or frightening. The item choice was guided by the Dunedin Study's age-11 interview protocol,^15^ an instrument prepared for the Avon Longitudinal Study of Parents and Children,^16^ and item pools since formalized in prodromal psychosis screening instruments^17^ including the Prevention through Risk Identification, Management and Education (PRIME)-screen,^18^ and the Structured Interview for Psychosis-Risk Syndromes (SIPS).^19^ Interviewers coded each of the 13 items (7 hallucination/delusion items plus 6 unusual experiences items) 0, 1, 2, indicating respectively “not present”, “probably present”, and “definitely present”. Responses to each of the 13 items (none, probable, definite) were summed to create a psychotic experiences scale (potential range=0–26, actual range=0–18, M=1.19, SD=2.58). The psychotic experiences measure did not involve clinical verification, meaning that this is a self-report measure capturing a broader range of mild, moderate, and potentially clinically pertinent hallucinations, delusions, and other unusual feelings and thoughts. Since there were low numbers of adolescents with high psychotic experiences scores (e.g., only 1.0% [N=21] of participants had a psychotic experiences score of 13 or more), scores were placed into an ordinal scale to tackle the skewed distribution while retaining more information than a binary score. Just over 30% of participants had at least one psychotic experience between ages 12 and 18: 69.8% reported no psychotic experiences (coded 0; N=1,440), 15.5% reported 1 or 2 psychotic experiences (coded 1; N=319), 8.1% reported 3–5 psychotic experiences (coded 2: N=166), and 6.7% reported 6 or more psychotic experiences (coded 3: N=138). This 30.2% prevalence is similar to the prevalence of self-reported psychotic experiences in other community samples of teenagers and young adults.^20-22^

*Inflammatory biomarkers.* Venous blood was collected from 1,700 of the 2,066 participants at age 18 (82.3%) with EDTA tubes. Tubes were spun at 2500g for 10 minutes and plasma samples obtained. Samples were stored at −80°C. Plasma samples were available for 1,448 participants. We examined three inflammatory biomarkers: C-reactive protein (CRP) and interleukin 6 (IL-6), which are the most studied inflammatory biomarkers; and a novel inflammatory biomarker, soluble urokinase plasminogen activator receptor (suPAR). Plasma CRP was measured using enzyme-linked immunosorbent assay (ELISA) (Quantikine ELISA Kit DCRP00, R&D Systems) following the manufacturer’s protocol. The coefficient of variation was 5.6%. Plasma IL-6 was measured using ELISA (Quantikine HS ELISA Kit HS600C, R&D Systems) following the manufacturer’s protocol. The coefficient of variation was 12.6%. Plasma suPAR levels were analyzed using ELISA (suPARnostic AUTO Flex ELISA, ViroGates A/S) following the manufacturer’s protocol. The coefficient of variation was 6%. CRP is a protein produced by the liver. IL-6 is a key cytokine with largely pro-inflammatory effects as well as regulatory role in the immune system. suPAR is a membrane protein. Both CRP and IL-6 are involved in the acute-phase response (e.g., infections), but may also reflect chronic inflammation. In contrast, suPAR can be considered a more stable marker of historic and chronic immune system activation since it is cleaved from membranes of immunologically active cells when inflammation is higher.^23^ CRP data were available for 1,430 participants, IL-6 data were available for 1,440 participants, and suPAR data were available for 1,444 participants.

*Covariates*

*Family psychiatric* history was assessed in private interviews with the mothers when children were aged 12. Mothers reported on their own mental health history and the mental health history of their biological mothers, fathers, sisters, brothers, and the twins’ biological father.^24^ This was converted into the proportion of family members with a history of psychiatric disorder (range=0-1).^25^

*Parental educational attainment* was measured in private interviews with the mothers when children were aged 5, taking the highest educational qualification achieved by either the twins’ mother or father (coded 0= CSE grades 0-5, GCSE grades A-G, A-level, HNC, HND, degree, postgraduate degree, 1=no qualifications).

*Genotyping participants.* We used Illumina HumanOmni Express 24 BeadChip arrays (Version 1.1; Illumina, Hayward, CA) to assay common single-nucleotide polymorphism (SNP) variation in the genomes of cohort members. The resulting database was restricted to SNPs called successfully in >98% of the cohort and in Hardy-Weinberg equilibrium (p>0.001). We imputed additional SNPs using the IMPUTE2 software (Version 2.3.1; [https://mathgen.stats.ox.ac.uk/ impute/impute_v2.html](https://mathgen.stats.ox.ac.uk/%20impute/impute_v2.html)^26^) and the 1000 Genomes Phase 3 reference panel (Genomes Project Consortium, 2015). Imputation was conducted on autosomal SNPs appearing in dbSNP (Version 140; [http://www.ncbi.nlm.nih.gov/SNP](http://www.ncbi.nlm.nih.gov/SNP/)/^27^) that were “called” in more than 98% of the samples. Invariant SNPs were excluded. The E-Risk cohort contains MZ twins, who are genetically identical; we therefore empirically measured genotypes of one randomly selected twin per pair and assigned these data to their MZ co-twin. We directly measured genotypes of both members of dizygotic (DZ) twin pairs. Prephasing and imputation were conducted using a 50-million-base-pair sliding window. The resulting genotype databases included genotyped SNPs and SNPs imputed with 90% probability of a specific genotype among the European descent members of the E-Risk cohort (N=1,999 participants in 1,011 families).

*Polygenic risk scores (PRS).* PRS were created for European descent E-Risk participants following the method described by Dudbridge^28^ using PRSice (Version 1.22; [http://prsice.info/](about:blank)).^29^ For schizophrenia PRS, SNPs reported in the results of the latest GWAS for schizophrenia^30^ were matched with SNPs in the E-Risk cohort, irrespective of nominal significance with schizophrenia. We then performed clumping by retaining the SNP with the smallest p-value from each LD block (excluding SNPs with *r*^2^ > 0.1 in 500-kb windows), then weighted retained SNPs by effect estimate. To control for possible population stratification, we conducted a principal component analysis of our genome-wide SNP database using PLINK (Version 1.9).^31^ One twin was selected at random from each family for principal component analysis. SNP loadings for principal components were applied to co-twin genetic data to compute principal component values for the full sample. The 10 principal components explained 2.8% of the variance in the schizophrenia PRS. We residualized polygenic scores for the first 10 principal components estimated from the genome-wide SNP data. The residualized scores were normally distributed. We standardized residuals for analysis (in the analytic sample, M=-0.02, SD=1, range=-3.75–3.53). PRS for educational attainment and cognitive performance were both obtained from the Social Science Genetic Association Consortium’s (SSGAC) Polygenic Risk Repository.^32^ PRS for educational attainment was derived from 3 separate GWAS (in the analytic sample, M=0.01, SD=1, range=-3.70–3.31).^33-35^ PRS for cognitive performance was derived from 2 separate GWAS (in the analytic sample, M=0.01, SD=1, range=-2.93–2.80).^36,37^ We used multi-trait^38^ versions of both educational attainment and cognitive performance PRS given that multi-trait PRS are generally considered to have higher predictive power than single-trait PRS.

*Multiple imputation by chained equations.* We imputed missing exposure and covariate data for those with complete psychotic experience and cognitive ability/inflammation data (Table 1) using “mi impute chained” in Stata v17.0. This was conducted separately for cognitive ability and inflammation models given that covariates differed between these models. Imputed variables included the physical and socioeconomic risk factor scores (or the separate socioenvironmental variables), family psychiatric history, PRS for schizophrenia, PRS for educational attainment, PRS for cognitive performance, and body temperature at age 18. Together with complete variables (psychotic experiences, cognitive abilities/inflammation, biological sex at birth, and parental education) we included an auxiliary variable describing baseline risk based on mother’s age. We imputed 5 datasets using a random seed of 1234. Imputation resulted in a sample size of N=2,006 for cognitive ability models (versus N=1,699-1,797 for complete case analyses) and a sample size of N=1,430 for inflammation models (versus N=1,199-1,230 for complete case analyses).

| Participants (%) |  |
| --- | --- |
|  | Index of Multiple Deprivation Deciles  (1=most deprived, 10=least deprived) |

**Supplementary Figure 1. Distribution of E-Risk participants at age 18 across Index of Multiple Deprivation deciles**

Note: This histogram shows E-Risk families’ addresses are a near-perfect match to the deciles of the UK’s 2015 Lower-layer Super Output Area (LSOA) Index of Multiple Deprivation (IMD) which averages 1,500 residents (or 650 households each); approximately 10% of the E-Risk cohort fills each of the IMD’s 10% bands, indicating that the E-Risk cohort accurately represents the distribution of deprivation in the UK.

**Supplementary Table 1. Performance statistics of CMAQ-Urban for 2004**

| **Pollutant** | **Number of data** | **Observed mean (µg/m^3^)** | **Modelled mean (µg/m^3^)** | **FAC2** | **MB (µg/m^3^)** | **NMB** | **RMSE (µg/m^3^)** | **r** |
| --- | --- | --- | --- | --- | --- | --- | --- | --- |
| NO_2_ | 184 | 42.70 | 41.67 | 0.98 | -1.02 | -0.02 | 13.49 | 0.78 |

Note: FAC2, fraction of predictions within a factor of two; MB, mean bias; NMB, normalized mean bias; NO_2_, nitrogen dioxide; RMSE, root mean squared error; r, correlation coefficient.

**Supplementary Table 2. Polychoric correlation matrix between socioenvironmental variables**

|  | Urbanicity | Air pollution | Deprivation | Disorder | Family disadvantage |
| --- | --- | --- | --- | --- | --- |
| Urbanicity |  |  |  |  |  |
| Air pollution | 0.75*** |  |  |  |  |
| Neighborhood deprivation | 0.33*** | 0.25*** |  |  |  |
| Neighborhood disorder | 0.14*** | 0.12*** | 0.31*** |  |  |
| Family disadvantage | 0.23*** | 0.21*** | 0.60*** | 0.38*** |  |

Note: ***P<0.001

**Supplementary Table 3. Variance explained by two-factor solution**

| **Factor** | **Variance** | **Difference** | **Proportion** | **Cumulative** |
| --- | --- | --- | --- | --- |
| Factor 1 – “Physical risk” | 1.38 | 0.18 | 0.65 | 0.65 |
| Factor 2 – “Socioeconomic risk” | 1.20 | - | 0.56 | 1.21 |

**Supplementary Table 4. Rotated factor loadings and uniqueness**

| **Variable** | **Factor 1 – “Physical risk”** | **Factor 1 – “Socioeconomic risk”** | **Uniqueness** |
| --- | --- | --- | --- |
| Urbanicity | 0.81 | 0.17 | 0.32 |
| Air pollution | 0.80 | 0.12 | 0.35 |
| Neighborhood deprivation | 0.24 | 0.67 | 0.50 |
| Neighborhood disorder | 0.09 | 0.45 | 0.79 |
| Family disadvantage | 0.15 | 0.71 | 0.48 |

Note: Grey cells have factor loadings >0.4

**Supplementary Figure 2. Scree plot from factor analysis**

**
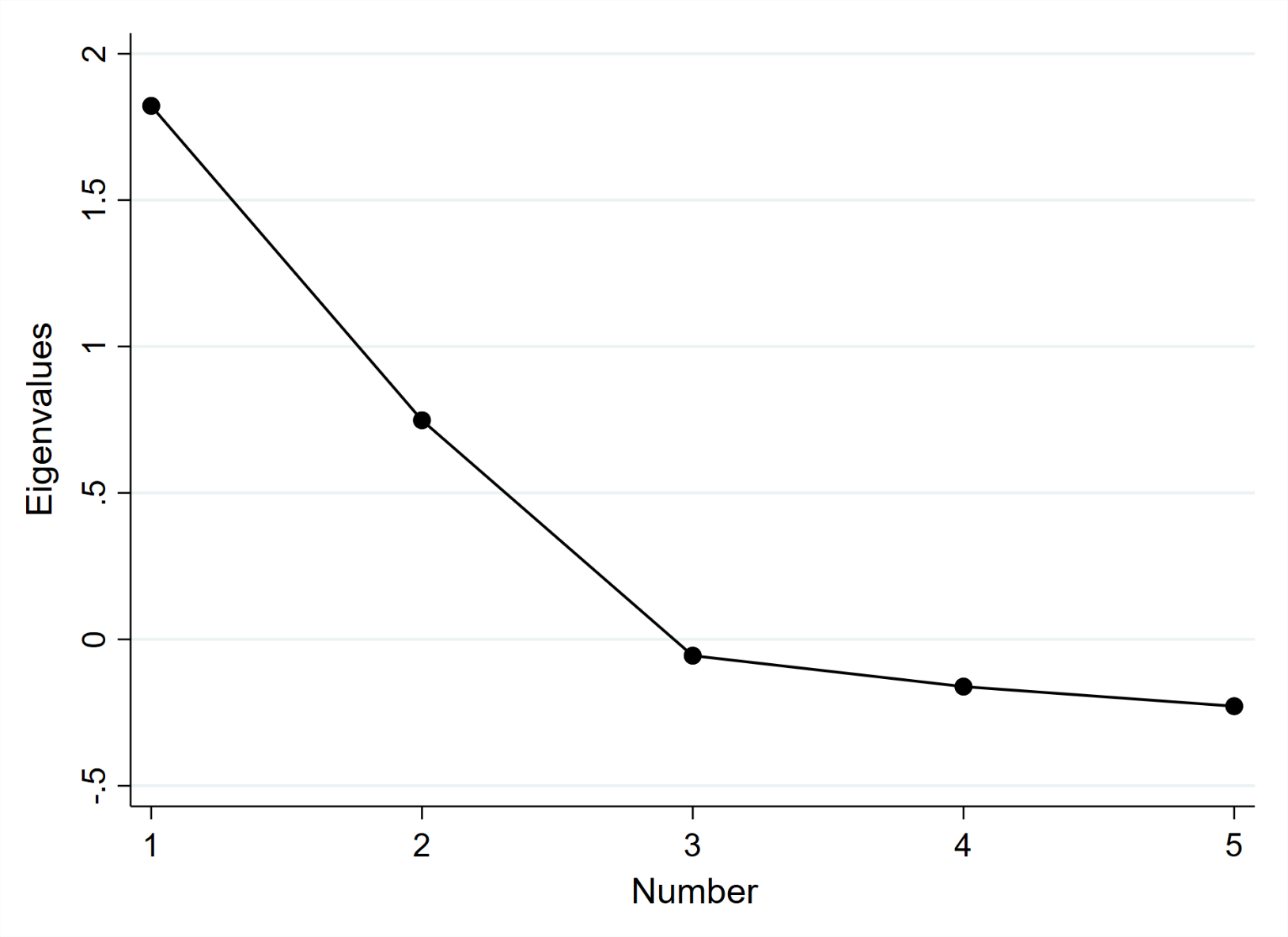
**

**Supplementary Table 5. Association of socioenvironmental adversity with cognitive ability and inflammation**

| **Mediator** | **Association of physical risk with cognitive ability and inflammation** | | | | **Association of socioeconomic risk with cognitive ability and inflammation** | | | |
| --- | --- | --- | --- | --- | --- | --- | --- | --- |
|  | **Unadjusted** | | **Adjusted** | | **Unadjusted** | | **Adjusted** | |
|  | **B**  **(95% CI)** | **P-value** | **B**  **(95% CI)** | **P-value** | **B**  **(95% CI)** | **P-value** | **B**  **(95% CI)** | **P-value** |
| Overall cognitive ability | -0.47  (-1.79 – 0.86) | 0.489 | -0.07  (-1.22 – 1.07) | 0.898 | -9.10  (-10.41 – -7.80) | <0.001 | -5.92  (-7.20 – -4.64) | <0.001 |
| Crystalized ability | -0.28  (-0.57 – 0.02) | 0.067 | -0.19  (-0.46 – 0.07) | 0.143 | -2.05  (-2.33 – -1.78) | <0.001 | -1.38  (-1.66 – -1.09) | <0.001 |
| Fluid ability | -0.08  (-0.32 – 0.15) | 0.485 | -0.02  (-0.24 – 0.21) | 0.870 | -1.24  (-1.48 – -1.00) | <0.001 | -0.82  (-1.07 – -0.57) | <0.001 |
| Working memory | 0.14  (-0.15 – 0.42) | 0.355 | 0.18  (-0.09 – 0.45) | 0.194 | -1.22  (-1.51 – -0.93) | <0.001 | -0.74  (-1.03 – -0.45) | <0.001 |
| CRP | -0.16  (-0.30 – -0.02) | 0.022 | -0.16  (-0.30 – -0.03) | 0.021 | 0.00  (-0.14 – 0.14) | 0.969 | -0.02  (-0.17 – 0.14) | 0.844 |
| IL-6 | -0.04  (-0.10 – 0.02) | 0.200 | -0.04  (-0.10 – 0.02) | 0.182 | 0.07  (0.01 – 0.13) | 0.014 | 0.07  (0.01 – 0.14) | 0.022 |
| suPAR | -0.09  (-0.18 – -0.00) | 0.039 | -0.10  (-0.19 – -0.01) | 0.030 | 0.21  (0.12 – 0.31) | <0.001 | 0.20  (0.10 – 0.31) | <0.001 |

Note: B=beta, CI=confidence interval, CRP=C-reactive protein, IL-6=interleukin-6, suPAR=soluble urokinase plasminogen activator receptor. All analyses were conducted following multiple imputation and control for the non-independence of twin observations. Model adjustments are described in the Methods and in Figure 2.

**Supplementary Table 6. Association of cognitive ability and inflammation with adolescent psychotic experiences**

| **Cognitive ability/inflammatory marker** | **Association with psychotic experiences** | | | |
| --- | --- | --- | --- | --- |
|  | **Unadjusted** | | **Adjusted** | |
|  | **OR (95% CI)** | **P-value** | **OR (95% CI)** | **P-value** |
| Overall cognitive ability | 0.98 (0.98 – 0.99) | <0.001 | 0.99 (0.98 – 0.99) | 0.001 |
| Crystalized ability | 0.91 (0.88 – 0.94) | <0.001 | 0.92 (0.89 – 0.96) | <0.001 |
| Fluid ability | 0.95 (0.92 – 0.99) | 0.014 | 0.98 (0.94 – 1.02) | 0.267 |
| Working memory | 0.95 (0.92 – 0.98) | 0.001 | 0.97 (0.93 – 1.00) | 0.038 |
| CRP | 1.02 (0.93 – 1.11) | 0.718 | 1.02 (0.93 – 1.12) | 0.655 |
| IL-6 | 0.98 (0.82 – 1.17) | 0.794 | 0.97 (0.81 – 1.16) | 0.764 |
| suPAR | 1.15 (1.00 – 1.33) | 0.049 | 1.18 (1.02 – 1.36) | 0.031 |

Note: CI=confidence interval, CRP=C-reactive protein, IL-6=interleukin-6, OR=odds ratio, suPAR=soluble urokinase plasminogen activator receptor. All analyses were conducted following multiple imputation and control for the non-independence of twin observations. Model adjustments are described in the Methods and in Figure 2.

**Supplementary Table 7.** **Mediation of the association between socioenvironmental adversity and adolescent psychotic experiences, via cognitive ability and inflammation**

| **Socioenvironmental adversity factor score** | **Total, direct, and indirect effects on adolescent psychotic experiences** | | | | | |
| --- | --- | --- | --- | --- | --- | --- |
| **Mediator** | **Unadjusted** | | | **Adjusted** | | |
|  | **OR (95% CI)** | **P-value** | **% Mediated*** | **OR (95% CI)** | **P-value** | **% Mediated*** |
| **Physical risk** |  |  |  |  |  |  |
| Overall cognitive ability |  |  |  |  |  |  |
| Total effect | 1.33 (1.10 – 1.57) | 0.001 |  | 1.32 (1.08 – 1.56) | 0.002 |  |
| Direct effect | 1.32 (1.09 – 1.56) | 0.002 |  | 1.32 (1.08 – 1.55) | 0.002 |  |
| Indirect effect via overall cognitive ability | 1.01 (0.98 – 1.03) | 0.490 | 2.82 | 1.00 (0.99 – 1.02) | 0.898 | 0.35 |
| Specific cognitive abilities |  |  |  |  |  |  |
| Total effect | 1.33 (1.10 – 1.57) | 0.002 |  | 1.32 (1.09 – 1.56) | 0.002 |  |
| Direct effect | 1.31 (1.07 – 1.54) | 0.003 |  | 1.31 (1.07 – 1.55) | 0.003 |  |
| Indirect effect via crystalized ability | 1.03 (1.00 – 1.06) | 0.093 | 8.67 | 1.02 (0.99 – 1.04) | 0.180 | 5.41 |
| Indirect effect via fluid ability | 1.00 (0.99 – 1.00) | 0.628 | NA | 1.00 (0.99 – 1.00) | 0.873 | NA |
| Indirect effect via working memory | 1.00 (0.99 – 1.01) | 0.458 | NA | 1.00 (0.99 – 1.00) | 0.444 | NA |
| Inflammatory markers |  |  |  |  |  |  |
| Total effect | 1.41 (1.14 – 1.73) | 0.001 |  | 1.41 (1.11 – 1.71) | 0.001 |  |
| Direct effect | 1.44 (1.15 – 1.73) | <0.001 |  | 1.44 (1.13 – 1.74) | 0.001 |  |
| Indirect effect via CRP | 1.00 (0.98 – 1.01) | 0.771 | NA | 1.00 (0.98 – 1.01) | 0.649 | NA |
| Indirect effect via IL-6 | 1.00 (0.99 – 1.01) | 0.461 | 0.89 | 1.00 (0.99 – 1.01) | 0.397 | 1.14 |
| Indirect effect via suPAR | 0.98 (0.96 – 1.00) | 0.119 | NA | 0.98 (0.96 – 1.00) | 0.100 | NA |
| **Socioeconomic risk** |  |  |  |  |  |  |
| Overall cognitive ability |  |  |  |  |  |  |
| Total effect | 1.78 (1.47 – 2.10) | <0.001 |  | 1.61 (1.29 – 1.93) | <0.001 |  |
| Direct effect | 1.62 (1.32 – 1.91) | <0.001 |  | 1.52 (1.21 – 1.83) | <0.001 |  |
| Indirect effect via overall cognitive ability | 1.10 (1.03 – 1.18) | 0.006 | 16.99 | 1.06 (1.01 – 1.11) | 0.023 | 11.47 |
| Specific cognitive abilities |  |  |  |  |  |  |
| Total effect | 1.79 (1.47 – 2.10) | <0.001 |  | 1.61 (1.29 – 1.93) | <0.001 |  |
| Direct effect | 1.57 (1.27 – 1.87) | <0.001 |  | 1.49 (1.18 – 1.79) | <0.001 |  |
| Indirect effect via crystalized ability | 1.15 (1.05 – 1.25) | 0.001 | 24.08 | 1.09 (1.03 – 1.16) | 0.004 | 18.94 |
| Indirect effect via fluid ability | 0.97 (0.92 – 1.02) | 0.255 | NA | 0.98 (0.95 – 1.01) | 0.240 | NA |
| Indirect effect via working memory | 1.02 (0.97 – 1.07) | 0.390 | 3.44 | 1.01 (0.98 – 1.04) | 0.485 | 2.14 |
| Inflammatory markers |  |  |  |  |  |  |
| Total effect | 1.81 (1.43 – 2.19) | <0.001 |  | 1.74 (1.34 – 2.15) | <0.001 |  |
| Direct effect | 1.78 (1.40 – 2.17) | <0.001 |  | 1.71 (1.31 – 2.12) | <0.001 |  |
| Indirect effect via CRP | 1.00 (1.00 – 1.00) | 0.969 | 0.01 | 1.00 (1.00 – 1.00) | 0.854 | NA |
| Indirect effect via IL-6 | 0.99 (0.97 – 1.01) | 0.308 | NA | 0.99 (0.97 – 1.01) | 0.297 | NA |
| Indirect effect via suPAR | 1.02 (0.99 – 1.06) | 0.182 | 3.87 | 1.03 (0.99 – 1.06) | 0.133 | 4.81 |

Note: CI=confidence interval, CRP=C-reactive protein, IL-6=interleukin-6, OR=odds ratio, suPAR=soluble urokinase receptor protein, *mediation percentages are marked NA (not applicable) where there is inconsistent mediation, i.e., the indirect effect OR is ≤1.00, leading to a negative percentage. All analyses were conducted following multiple imputation and control for the non-independence of twin observations. Model adjustments are described in the Methods and in Figure 2. For mediation models, overall cognitive ability was included as a separate mediator. In contrast, specific cognitive abilities were included simultaneously to account for potentially overlapping pathways. Likewise, inflammatory markers were included simultaneously. Note that total effects differ slightly across models due to differences in model specifications and sample sizes.

**Supplementary Table 8. Association of urbanicity with cognitive ability and inflammation**

| **Mediator** | **Association of urbanicity with cognitive ability and inflammation** | | | |
| --- | --- | --- | --- | --- |
|  | **Unadjusted** | | **Adjusted** | |
|  | **B (95% CI)** | **P-value** | **B (95% CI)** | **P-value** |
| Overall cognitive ability #1* | -2.65 (-4.88 - -0.42) | 0.020 | -2.04 (-3.95 - -0.14) | 0.036 |
| Overall cognitive ability #2* | -2.61 (-5.02 - -0.20) | 0.034 | -1.23 (-3.32 - 0.87) | 0.251 |
| Crystalized ability #1 | -0.87 (-1.36 - -0.39) | <0.001 | -0.72 (-1.15 - -0.29) | 0.001 |
| Crystalized ability #2 | -0.94 (-1.46 - -0.42) | <0.001 | -0.64 (-1.10 - -0.18) | 0.007 |
| Fluid ability #1 | -0.21 (-0.61 - 0.19) | 0.301 | -0.15 (-0.51 - 0.22) | 0.438 |
| Fluid ability #2 | -0.34 (-0.77 - 0.09) | 0.122 | -0.14 (-0.53 - 0.25) | 0.486 |
| Working memory #1 | -0.22 (-0.69 - 0.24) | 0.349 | -0.14 (-0.58 - 0.29) | 0.516 |
| Working memory #2 | -0.01 (-0.52 - 0.50) | 0.974 | 0.18 (-0.30 - 0.65) | 0.471 |
| CRP #1 | -0.11 (-0.33 - 0.11) | 0.322 | -0.12 (-0.34 - 0.10) | 0.294 |
| CRP #2 | -0.22 (-0.47 - 0.03) | 0.078 | -0.22 (-0.47 - 0.02) | 0.073 |
| IL-6 #1 | 0.04 (-0.06 - 0.13) | 0.444 | 0.03 (-0.06 - 0.12) | 0.462 |
| IL-6 #2 | -0.03 (-0.13 - 0.07) | 0.520 | -0.04 (-0.13 - 0.06) | 0.481 |
| suPAR #1 | -0.04 (-0.20 - 0.11) | 0.587 | -0.05 (-0.21 - 0.11) | 0.520 |
| suPAR #2 | -0.20 (-0.36 - -0.03) | 0.020 | -0.21 (-0.38 - -0.05) | 0.012 |

Note: B=beta, CI=confidence interval, CRP=C-reactive protein, IL-6=interleukin-6, suPAR=soluble urokinase plasminogen activator receptor. *#1 is the associations for the middle versus bottom level of urbanicity. #2 is the associations for the top versus bottom level of urbanicity. All analyses were conducted following multiple imputation and control for the non-independence of twin observations. Model adjustments are described in the Methods and in Figure 2.

**Supplementary Table 9. Association of air pollution with cognitive ability and inflammation**

| **Mediator** | **Association of air pollution with cognitive ability and inflammation** | | | |
| --- | --- | --- | --- | --- |
|  | **Unadjusted** | | **Adjusted** | |
|  | **B (95% CI)** | **P-value** | **B (95% CI)** | **P-value** |
| Overall cognitive ability #1* | 0.51 (-1.53 - 2.56) | 0.621 | 0.49 (-1.31 - 2.30) | 0.592 |
| Overall cognitive ability #2* | -1.14 (-3.19 - 0.92) | 0.278 | -0.31 (-2.11 - 1.48) | 0.731 |
| Crystalized ability #1 | 0.08 (-0.36 - 0.52) | 0.717 | 0.07 (-0.33 - 0.46) | 0.741 |
| Crystalized ability #2 | -0.53 (-0.98 - -0.09) | 0.019 | -0.36 (-0.76 - 0.04) | 0.079 |
| Fluid ability #1 | 0.08 (-0.30 - 0.46) | 0.673 | 0.10 (-0.26 - 0.46) | 0.596 |
| Fluid ability #2 | -0.13 (-0.49 - 0.23) | 0.485 | -0.00 (-0.34 - 0.33) | 0.990 |
| Working memory #1 | 0.09 (-0.33 - 0.52) | 0.670 | 0.08 (-0.32 - 0.48) | 0.686 |
| Working memory #2 | 0.10 (-0.34 - 0.54) | 0.651 | 0.21 (-0.21 - 0.62) | 0.328 |
| CRP #1 | -0.17 (-0.38 - 0.03) | 0.095 | -0.17 (-0.38 - 0.03) | 0.099 |
| CRP #2 | -0.24 (-0.45 - -0.03) | 0.026 | -0.24 (-0.45 - -0.03) | 0.024 |
| IL-6 #1 | -0.00 (-0.09 - 0.08) | 0.938 | -0.01 (-0.09 - 0.08) | 0.897 |
| IL-6 #2 | -0.04 (-0.13 - 0.05) | 0.393 | -0.04 (-0.13 - 0.05) | 0.367 |
| suPAR #1 | 0.08 (-0.06 - 0.22) | 0.262 | 0.07 (-0.07 - 0.21) | 0.307 |
| suPAR #2 | -0.02 (-0.17 - 0.12) | 0.736 | -0.03 (-0.17 - 0.11) | 0.680 |

Note: B=beta, CI=confidence interval, CRP=C-reactive protein, IL-6=interleukin-6, suPAR=soluble urokinase plasminogen activator receptor. *#1 is the associations for the middle versus bottom level of air pollution. #2 is the associations for the top versus bottom level of air pollution. All analyses were conducted following multiple imputation and control for the non-independence of twin observations. Model adjustments are described in the Methods and in Figure 2.

**Supplementary Table 10. Association of neighborhood deprivation with cognitive ability and inflammation**

| **Mediator** | **Association of neighborhood deprivation with cognitive ability and inflammation** | | | |
| --- | --- | --- | --- | --- |
|  | **Unadjusted** | | **Adjusted** | |
|  | **B (95% CI)** | **P-value** | **B (95% CI)** | **P-value** |
| Overall cognitive ability #1* | -6.88 (-8.75 - -5.01) | <0.001 | -4.86 (-6.54 - -3.18) | <0.001 |
| Overall cognitive ability #2* | -11.98 (-14.07 - -9.90) | <0.001 | -7.38 (-9.35 - -5.40) | <0.001 |
| Crystalized ability #1 | -1.57 (-2.00 - -1.15) | <0.001 | -1.16 (-1.55 - -0.77) | <0.001 |
| Crystalized ability #2 | -2.68 (-3.12 - -2.23) | <0.001 | -1.70 (-2.13 - -1.28) | <0.001 |
| Fluid ability #1 | -0.97 (-1.30 - -0.63) | <0.001 | -0.70 (-1.02 - -0.38) | <0.001 |
| Fluid ability #2 | -1.67 (-2.04 - -1.30) | <0.001 | -1.07 (-1.43 - -0.70) | <0.001 |
| Working memory #1 | -0.86 (-1.26 - -0.46) | <0.001 | -0.54 (-0.92 - -0.16) | 0.005 |
| Working memory #2 | -1.59 (-2.06 - -1.12) | <0.001 | -0.89 (-1.36 - -0.42) | <0.001 |
| CRP #1 | -0.01 (-0.21 - 0.19) | 0.913 | -0.02 (-0.22 - 0.18) | 0.841 |
| CRP #2 | -0.10 (-0.33 - 0.14) | 0.420 | -0.11 (-0.36 - 0.13) | 0.354 |
| IL-6 #1 | 0.03 (-0.06 - 0.11) | 0.536 | 0.02 (-0.06 - 0.11) | 0.599 |
| IL-6 #2 | 0.03 (-0.07 - 0.13) | 0.540 | 0.02 (-0.08 - 0.13) | 0.699 |
| suPAR #1 | 0.14 (0.00 - 0.27) | 0.047 | 0.12 (-0.01 - 0.25) | 0.076 |
| suPAR #2 | 0.16 (0.01 - 0.31) | 0.034 | 0.13 (-0.03 - 0.28) | 0.104 |

Note: B=beta, CI=confidence interval, CRP=C-reactive protein, IL-6=interleukin-6, suPAR=soluble urokinase plasminogen activator receptor. *#1 is the associations for the middle versus bottom level of neighborhood deprivation. #2 is the associations for the top versus bottom level of neighborhood deprivation. All analyses were conducted following multiple imputation and control for the non-independence of twin observations. Model adjustments are described in the Methods and in Figure 2.

**Supplementary Table 11. Association of neighborhood disorder with cognitive ability and inflammation**

| **Mediator** | **Association of neighborhood disorder with cognitive ability and inflammation** | | | |
| --- | --- | --- | --- | --- |
|  | **Unadjusted** | | **Adjusted** | |
|  | **B (95% CI)** | **P-value** | **B (95% CI)** | **P-value** |
| Overall cognitive ability #1* | -1.13 (-3.16 - 0.89) | 0.273 | 0.25 (-1.52 - 2.03) | 0.780 |
| Overall cognitive ability #2* | -5.14 (-7.20 - -3.08) | <0.001 | -2.52 (-4.38 - -0.66) | 0.008 |
| Crystalized ability #1 | -0.29 (-0.75 - 0.16) | 0.209 | 0.01 (-0.40 - 0.41) | 0.964 |
| Crystalized ability #2 | -1.14 (-1.59 - -0.68) | <0.001 | -0.56 (-0.99 - -0.14) | 0.009 |
| Fluid ability #1 | -0.16 (-0.53 - 0.20) | 0.379 | 0.01 (-0.33 - 0.35) | 0.954 |
| Fluid ability #2 | -0.70 (-1.06 - -0.33) | <0.001 | -0.35 (-0.70 - -0.00) | 0.047 |
| Working memory #1 | -0.08 (-0.51 - 0.34) | 0.696 | 0.12 (-0.27 - 0.52) | 0.539 |
| Working memory #2 | -0.71 (-1.14 - -0.27) | 0.001 | -0.33 (-0.74 - 0.09) | 0.124 |
| CRP #1 | 0.00 (-0.20 - 0.21) | 0.965 | -0.00 (-0.21 - 0.20) | 0.976 |
| CRP #2 | 0.04 (-0.17 - 0.25) | 0.694 | 0.03 (-0.18 - 0.24) | 0.778 |
| IL-6 #1 | -0.04 (-0.13 - 0.05) | 0.382 | -0.05 (-0.14 - 0.05) | 0.333 |
| IL-6 #2 | 0.02 (-0.07 - 0.11) | 0.722 | 0.01 (-0.08 - 0.10) | 0.842 |
| suPAR #1 | 0.09 (-0.05 - 0.23) | 0.212 | 0.07 (-0.06 - 0.21) | 0.296 |
| suPAR #2 | 0.24 (0.10 - 0.38) | 0.001 | 0.22 (0.07 - 0.36) | 0.003 |

Note: B=beta, CI=confidence interval, CRP=C-reactive protein, IL-6=interleukin-6, suPAR=soluble urokinase plasminogen activator receptor. *#1 is the associations for the middle versus bottom level of neighborhood disorder. #2 is the associations for the top versus bottom level of neighborhood disorder. All analyses were conducted following multiple imputation and control for the non-independence of twin observations. Model adjustments are described in the Methods and in Figure 2.

**Supplementary Table 12. Association of family disadvantage with cognitive ability and inflammation**

| **Outcome/mediator** | **Association of family disadvantage with cognitive ability and inflammation** | | | |
| --- | --- | --- | --- | --- |
|  | **Unadjusted** | | **Adjusted** | |
|  | **B (95% CI)** | **P-value** | **B (95% CI)** | **P-value** |
| Overall cognitive ability #1* | -5.46 (-7.27 - -3.65) | <0.001 | -3.61 (-5.26 - -1.96) | <0.001 |
| Overall cognitive ability #2* | -12.39 (-14.39 - -10.40) | <0.001 | -7.87 (-9.85 - -5.89) | <0.001 |
| Crystalized ability #1 | -1.53 (-1.93 - -1.13) | <0.001 | -1.15 (-1.52 - -0.77) | <0.001 |
| Crystalized ability #2 | -2.82 (-3.24 - -2.41) | <0.001 | -1.87 (-2.31 - -1.44) | <0.001 |
| Fluid ability #1 | -0.70 (-1.03 - -0.37) | <0.001 | -0.46 (-0.78 - -0.15) | 0.004 |
| Fluid ability #2 | -1.67 (-2.04 - -1.30) | <0.001 | -1.06 (-1.45 - -0.68) | <0.001 |
| Working memory #1 | -0.49 (-0.89 - -0.09) | 0.017 | -0.19 (-0.58 - 0.19) | 0.322 |
| Working memory #2 | -1.64 (-2.07 - -1.21) | <0.001 | -0.96 (-1.40 - -0.53) | <0.001 |
| CRP #1 | -0.04 (-0.24 - 0.16) | 0.675 | -0.06 (-0.26 - 0.14) | 0.581 |
| CRP #2 | 0.02 (-0.20 - 0.24) | 0.837 | -0.00 (-0.24 - 0.23) | 0.985 |
| IL-6 #1 | 0.05 (-0.04 - 0.14) | 0.270 | 0.05 (-0.04 - 0.14) | 0.249 |
| IL-6 #2 | 0.15 (0.06 - 0.25) | 0.002 | 0.16 (0.06 - 0.26) | 0.002 |
| suPAR #1 | 0.24 (0.10 - 0.38) | 0.001 | 0.23 (0.09 - 0.37) | 0.001 |
| suPAR #2 | 0.30 (0.15 - 0.45) | <0.001 | 0.29 (0.13 - 0.45) | <0.001 |

Note: B=beta, CI=confidence interval, CRP=C-reactive protein, IL-6=interleukin-6, suPAR=soluble urokinase plasminogen activator receptor. *#1 is the associations for the middle versus bottom level of family disadvantage. #2 is the associations for the top versus bottom level of family disadvantage. All analyses were conducted following multiple imputation and control for the non-independence of twin observations. Model adjustments are described in the Methods and in Figure 2.

**Supplementary Table 13. Association between urbanicity and adolescent psychotic experiences, mediated via cognitive ability**

| **Cognitive ability mediator** | **Associations split into total, direct and indirect effects and mediation percentage** | | | | | |
| --- | --- | --- | --- | --- | --- | --- |
|  | **Unadjusted** | | | **Adjusted** | | |
|  | **OR (95% CI)** | **P-value** | **% Mediated*** | **OR (95% CI)** | **P-value** | **% Mediated*** |
| Overall cognitive ability |  |  |  |  |  |  |
| Total effect #1** | 1.14 (0.79 – 1.49) | 0.408 |  | 1.08 (0.75 – 1.41) | 0.626 |  |
| Total effect #2** | 1.56 (1.05 – 2.07) | 0.007 |  | 1.48 (1.00 – 1.96) | 0.019 |  |
| Direct effect #1 | 1.09 (0.75 – 1.42) | 0.589 |  | 1.05 (0.73 – 1.38) | 0.750 |  |
| Direct effect #2 | 1.49 (1.01 – 1.97) | 0.015 |  | 1.46 (0.98 – 1.93) | 0.023 |  |
| Indirect effect via overall cognitive ability #1 | 1.05 (1.00 – 1.09) | 0.036 | 35.13 | 1.03 (1.00 – 1.06) | 0.072 | 34.73 |
| Indirect effect via overall cognitive ability #2 | 1.05 (1.00 – 1.09) | 0.049 | 10.19 | 1.02 (0.99 – 1.05) | 0.271 | 4.07 |
| Specific cognitive abilities |  |  |  |  |  |  |
| Total effect #1 | 1.14 (0.79 – 1.50) | 0.406 |  | 1.08 (0.75 – 1.42) | 0.622 |  |
| Total effect #2 | 1.56 (1.05 – 2.07) | 0.008 |  | 1.49 (1.00 – 1.98) | 0.018 |  |
| Direct effect #1 | 1.05 (0.73 – 1.38) | 0.748 |  | 1.02 (0.70 – 1.34) | 0.894 |  |
| Direct effect #2 | 1.44 (0.97 – 1.91) | 0.028 |  | 1.42 (0.95 – 1.89) | 0.037 |  |
| Indirect effect via crystalized ability #1 | 1.08 (1.02 – 1.14) | 0.005 | 60.22 | 1.06 (1.01 – 1.11) | 0.014 | 73.58 |
| Indirect effect via crystalized ability #2 | 1.09 (1.02 – 1.15) | 0.006 | 19.16 | 1.05 (1.00 – 1.10) | 0.032 | 12.88 |
| Indirect effect via fluid ability #1 | 1.00 (0.99 – 1.01) | 0.549 | NA | 1.00 (0.99 – 1.01) | 0.547 | NA |
| Indirect effect via fluid ability #2 | 0.99 (0.98 – 1.01) | 0.510 | NA | 1.00 (0.99 – 1.01) | 0.580 | NA |
| Indirect effect via working memory #1 | 1.01 (0.99 – 1.02) | 0.470 | 3.82 | 1.00 (0.99 – 1.01) | 0.598 | 3.27 |
| Indirect effect via working memory #2 | 1.00 (0.99 – 1.01) | 0.974 | 0.04 | 1.00 (0.99 – 1.01) | 0.563 | NA |

Note: CI=confidence interval, OR=odds ratio, *mediation percentages are marked NA (not applicable) where there is inconsistent mediation, i.e., the indirect effect OR is ≤1.00, leading to a negative percentage, ** #1 is the associations for the middle versus bottom level of urbanicity. #2 is the associations for the top versus bottom level of urbanicity. All analyses were conducted following multiple imputation and control for the non-independence of twin observations. Model adjustments are described in the Methods and in Figure 2.

**Supplementary Table 14. Association between air pollution and adolescent psychotic experiences, mediated via cognitive ability**

| **Cognitive ability mediator** | **Associations split into total, direct and indirect effects and mediation percentage** | | | | | |
| --- | --- | --- | --- | --- | --- | --- |
|  | **Unadjusted** | | | **Adjusted** | | |
|  | **OR (95% CI)** | **P-value** | **% Mediated*** | **OR (95% CI)** | **P-value** | **% Mediated*** |
| Overall cognitive ability |  |  |  |  |  |  |
| Total effect #1** | 1.01 (0.73 – 1.30) | 0.925 |  | 1.04 (0.75 – 1.33) | 0.771 |  |
| Total effect #2** | 1.63 (1.20 – 2.06) | <0.001 |  | 1.57 (1.16 – 1.98) | 0.001 |  |
| Direct effect #1 | 1.02 (0.74 – 1.31) | 0.875 |  | 1.05 (0.76 – 1.34) | 0.736 |  |
| Direct effect #2 | 1.60 (1.18 – 2.01) | <0.001 |  | 1.57 (1.16 – 1.97) | 0.001 |  |
| Indirect effect via overall cognitive ability #1 | 0.99 (0.96 – 1.03) | 0.621 | NA | 0.99 (0.97 – 1.02) | 0.598 | NA |
| Indirect effect via overall cognitive ability #2 | 1.02 (0.98 – 1.06) | 0.282 | 3.98 | 1.00 (0.98 – 1.03) | 0.730 | 0.89 |
| Specific cognitive abilities |  |  |  |  |  |  |
| Total effect #1 | 1.01 (0.73 – 1.30) | 0.930 |  | 1.04 (0.75 – 1.33) | 0.771 |  |
| Total effect #2 | 1.63 (1.20 – 2.06) | <0.001 |  | 1.58 (1.17 – 1.99) | 0.001 |  |
| Direct effect #1 | 1.02 (0.74 – 1.30) | 0.883 |  | 1.05 (0.76 – 1.34) | 0.744 |  |
| Direct effect #2 | 1.56 (1.15 – 1.97) | 0.001 |  | 1.54 (1.14 – 1.95) | 0.001 |  |
| Indirect effect via crystalized ability #1 | 0.99 (0.95 – 1.03) | 0.716 | NA | 0.99 (0.97 – 1.02) | 0.740 | NA |
| Indirect effect via crystalized ability #2 | 1.05 (1.00 – 1.09) | 0.044 | 9.52 | 1.03 (0.99 – 1.06) | 0.126 | 6.03 |
| Indirect effect via fluid ability #1 | 1.00 (0.99 – 1.01) | 0.725 | 9.52 | 1.00 (0.99 – 1.01) | 0.648 | 4.71 |
| Indirect effect via fluid ability #2 | 1.00 (0.99 – 1.01) | 0.627 | NA | 1.00 (0.99 – 1.01) | 0.990 | NA |
| Indirect effect via working memory #1 | 1.00 (0.99 – 1.01) | 0.690 | NA | 1.00 (0.99 – 1.01) | 0.714 | NA |
| Indirect effect via working memory #2 | 1.00 (0.99 – 1.01) | 0.670 | NA | 1.00 (0.99 – 1.01) | 0.495 | NA |

Note: CI=confidence interval, OR=odds ratio, *mediation percentages are marked NA (not applicable) where there is inconsistent mediation, i.e., the indirect effect OR is ≤1.00, leading to a negative percentage, ** #1 is the associations for the middle versus bottom level of air pollution. #2 is the associations for the top versus bottom level of air pollution. All analyses were conducted following multiple imputation and control for the non-independence of twin observations. Model adjustments are described in the Methods and in Figure 2.

**Supplementary Table 15. Association between neighborhood deprivation and adolescent psychotic experiences, mediated via cognitive ability**

| **Cognitive ability mediator** | **Associations split into total, direct and indirect effects and mediation percentage** | | | | | |
| --- | --- | --- | --- | --- | --- | --- |
|  | **Unadjusted** | | | **Adjusted** | | |
|  | **OR (95% CI)** | **P-value** | **% Mediated*** | **OR (95% CI)** | **P-value** | **% Mediated*** |
| Overall cognitive ability |  |  |  |  |  |  |
| Total effect #1** | 1.45 (1.07 – 1.83) | 0.005 |  | 1.36 (1.00 – 1.72) | 0.023 |  |
| Total effect #2** | 2.00 (1.43 – 2.57) | <0.001 |  | 1.72 (1.20 – 2.25) | <0.001 |  |
| Direct effect #1 | 1.32 (0.97 – 1.67) | 0.042 |  | 1.29 (0.95 – 1.63) | 0.061 |  |
| Direct effect #2 | 1.70 (1.20 – 2.20) | <0.001 |  | 1.59 (1.10 – 2.08) | 0.003 |  |
| Indirect effect via overall cognitive ability #1 | 1.10 (1.04 – 1.16) | 0.001 | 25.52 | 1.05 (1.01 – 1.10) | 0.012 | 16.94 |
| Indirect effect via overall cognitive ability #2 | 1.18 (1.07 – 1.29) | <0.001 | 23.73 | 1.08 (1.02 – 1.15) | 0.010 | 14.45 |
| Specific cognitive abilities |  |  |  |  |  |  |
| Total effect #1 | 1.45 (1.07 – 1.83) | 0.006 |  | 1.35 (1.00 – 1.71) | 0.025 |  |
| Total effect #2 | 2.01 (1.43 – 2.59) | <0.001 |  | 1.73 (1.20 – 2.26) | <0.001 |  |
| Direct effect #1 | 1.28 (0.94 – 1.63) | 0.069 |  | 1.26 (0.92 – 1.59) | 0.098 |  |
| Direct effect #2 | 1.63 (1.15 – 2.12) | 0.001 |  | 1.55 (1.07 – 2.03) | 0.006 |  |
| Indirect effect via crystalized ability #1 | 1.13 (1.05 – 1.22) | <0.001 | 34.06 | 1.09 (1.03 – 1.15) | 0.004 | 27.83 |
| Indirect effect via crystalized ability #2 | 1.24 (1.10 – 1.38) | <0.001 | 30.78 | 1.13 (1.04 – 1.22) | 0.002 | 22.49 |
| Indirect effect via fluid ability #1 | 0.98 (0.94 – 1.02) | 0.340 | NA | 0.98 (0.95 – 1.01) | 0.275 | NA |
| Indirect effect via fluid ability #2 | 0.97 (0.90 – 1.03) | 0.336 | NA | 0.98 (0.93 – 1.02) | 0.271 | NA |
| Indirect effect via working memory #1 | 1.02 (0.98 – 1.05) | 0.367 | 4.03 | 1.01 (0.99 – 1.03) | 0.481 | 2.51 |
| Indirect effect via working memory #2 | 1.03 (0.97 – 1.09) | 0.365 | 3.94 | 1.01 (0.98 – 1.05) | 0.480 | 2.28 |

Note: CI=confidence interval, OR=odds ratio, *mediation percentages are marked NA (not applicable) where there is inconsistent mediation, i.e., the indirect effect OR is ≤1.00, leading to a negative percentage, ** #1 is the associations for the middle versus bottom level of neighborhood deprivation. #2 is the associations for the top versus bottom level of neighborhood deprivation. All analyses were conducted following multiple imputation and control for the non-independence of twin observations. Model adjustments are described in the Methods and in Figure 2.

**Supplementary Table 16. Association between neighborhood disorder and adolescent psychotic experiences, mediated via cognitive ability**

| **Cognitive ability mediator** | **Associations split into total, direct and indirect effects and mediation percentage** | | | | | |
| --- | --- | --- | --- | --- | --- | --- |
|  | **Unadjusted** | | | **Adjusted** | | |
|  | **OR (95% CI)** | **P-value** | **% Mediated*** | **OR (95% CI)** | **P-value** | **% Mediated*** |
| Overall cognitive ability |  |  |  |  |  |  |
| Total effect #1** | 1.34 (0.97 – 1.70) | 0.039 |  | 1.26 (0.92 – 1.61) | 0.094 |  |
| Total effect #2** | 1.68 (1.22 – 2.14) | <0.001 |  | 1.48 (1.07 – 1.90) | 0.006 |  |
| Direct effect #1 | 1.31 (0.95 – 1.67) | 0.052 |  | 1.27 (0.92 – 1.61) | 0.090 |  |
| Direct effect #2 | 1.55 (1.13 – 1.96) | 0.002 |  | 1.44 (1.04 – 1.83) | 0.011 |  |
| Indirect effect via overall cognitive ability #1 | 1.02 (0.98 – 1.05) | 0.281 | 6.46 | 1.00 (0.97 – 1.02) | 0.782 | NA |
| Indirect effect via overall cognitive ability #2 | 1.09 (1.04 – 1.14) | 0.001 | 16.30 | 1.03 (1.00 – 1.06) | 0.035 | 8.11 |
| Specific cognitive abilities |  |  |  |  |  |  |
| Total effect #1 | 1.34 (0.97 – 1.71) | 0.038 |  | 1.27 (0.92 – 1.62) | 0.092 |  |
| Total effect #2 | 1.69 (1.23 – 2.16) | <0.001 |  | 1.49 (1.07 – 1.91) | 0.005 |  |
| Direct effect #1 | 1.30 (0.95 – 1.66) | 0.056 |  | 1.27 (0.92 – 1.62) | 0.088 |  |
| Direct effect #2 | 1.52 (1.11 – 1.94) | 0.002 |  | 1.43 (1.03 – 1.83) | 0.012 |  |
| Indirect effect via crystalized ability #1 | 1.03 (0.98 – 1.07) | 0.221 | 9.00 | 1.00 (0.97 – 1.03) | 0.964 | NA |
| Indirect effect via crystalized ability #2 | 1.11 (1.04 – 1.17) | 0.001 | 19.43 | 1.05 (1.00 – 1.09) | 0.027 | 11.40 |
| Indirect effect via fluid ability #1 | 1.00 (0.99 – 1.01) | 0.607 | NA | 1.00 (0.99 – 1.01) | 0.954 | 0.08 |
| Indirect effect via fluid ability #2 | 0.99 (0.96 – 1.02) | 0.524 | NA | 0.99 (0.98 – 1.01) | 0.443 | NA |
| Indirect effect via working memory #1 | 1.00 (0.99 – 1.01) | 0.723 | 0.49 | 1.00 (0.99 – 1.01) | 0.652 | NA |
| Indirect effect via working memory #2 | 1.01 (0.98 – 1.04) | 0.390 | 2.28 | 1.00 (0.99 – 1.02) | 0.549 | 1.04 |

Note: CI=confidence interval, OR=odds ratio, *mediation percentages are marked NA (not applicable) where there is inconsistent mediation, i.e., the indirect effect OR is ≤1.00, leading to a negative percentage. ** #1 is the associations for the middle versus bottom level of neighborhood disorder. #2 is the associations for the top versus bottom level of neighborhood disorder. All analyses were conducted following multiple imputation and control for the non-independence of twin observations. Model adjustments are described in the Methods and in Figure 2.

**Supplementary Table 17. Association between family disadvantage and adolescent psychotic experiences, mediated via cognitive ability**

| **Cognitive ability mediator** | **Associations split into total, direct and indirect effects and mediation percentage** | | | | | |
| --- | --- | --- | --- | --- | --- | --- |
|  | **Unadjusted** | | | **Adjusted** | | |
|  | **OR (95% CI)** | **P-value** | **% Mediated*** | **OR (95% CI)** | **P-value** | **% Mediated*** |
| Overall cognitive ability |  |  |  |  |  |  |
| Total effect #1** | 1.72 (1.29 – 2.15) | <0.001 |  | 1.56 (1.16 – 1.96) | 0.001 |  |
| Total effect #2** | 2.18 (1.60 – 2.76) | <0.001 |  | 1.83 (1.28 – 2.37) | <0.001 |  |
| Direct effect #1 | 1.61 (1.21 – 2.01) | <0.001 |  | 1.50 (1.12 – 1.88) | 0.002 |  |
| Direct effect #2 | 1.88 (1.35 – 2.40) | <0.001 |  | 1.69 (1.18 – 2.20) | 0.001 |  |
| Indirect effect via overall cognitive ability #1 | 1.07 (1.02 – 1.12) | 0.004 | 12.12 | 1.04 (1.00 – 1.07) | 0.026 | 8.15 |
| Indirect effect via overall cognitive ability #2 | 1.16 (1.05 – 1.27) | 0.002 | 19.14 | 1.08 (1.01 – 1.15) | 0.016 | 13.07 |
| Specific cognitive abilities |  |  |  |  |  |  |
| Total effect #1 | 1.72 (1.29 – 2.15) | <0.001 |  | 1.56 (1.16 – 1.96) | 0.001 |  |
| Total effect #2 | 2.18 (1.59 – 2.76) | <0.001 |  | 1.83 (1.28 – 2.37) | <0.001 |  |
| Direct effect #1 | 1.56 (1.16 – 1.95) | 0.001 |  | 1.46 (1.08 – 1.83) | 0.004 |  |
| Direct effect #2 | 1.80 (1.29 – 2.31) | <0.001 |  | 1.63 (1.14 – 2.12) | 0.002 |  |
| Indirect effect via crystalized ability #1 | 1.11 (1.04 – 1.19) | 0.002 | 19.39 | 1.08 (1.02 – 1.14) | 0.007 | 16.96 |
| Indirect effect via crystalized ability #2 | 1.21 (1.07 – 1.36) | 0.001 | 24.92 | 1.13 (1.04 – 1.23) | 0.004 | 20.44 |
| Indirect effect via fluid ability #1 | 0.99 (0.96 – 1.01) | 0.337 | NA | 0.99 (0.97 – 1.01) | 0.316 | NA |
| Indirect effect via fluid ability #2 | 0.97 (0.90 – 1.03) | 0.324 | NA | 0.98 (0.93 – 1.02) | 0.294 | NA |
| Indirect effect via working memory #1 | 1.01 (0.99 – 1.03) | 0.344 | 1.77 | 1.00 (0.99 – 1.01) | 0.532 | 0.69 |
| Indirect effect via working memory #2 | 1.03 (0.97 – 1.10) | 0.310 | 4.13 | 1.02 (0.98 – 1.05) | 0.432 | 2.52 |

Note: CI=confidence interval, OR=odds ratio, *mediation percentages are marked NA (not applicable) where there is inconsistent mediation, i.e., the indirect effect OR is ≤1.00, leading to a negative percentage, ** #1 is the associations for the middle versus bottom level of family disadvantage. #2 is the associations for the top versus bottom level of family disadvantage. All analyses were conducted following multiple imputation and control for the non-independence of twin observations. Model adjustments are described in the Methods and in Figure 2.

**Supplementary Table 18. Association between urbanicity and adolescent psychotic experiences, mediated via inflammation**

| **Inflammatory marker mediator** | **Associations split into total, direct and indirect effects and mediation percentage** | | | | | |
| --- | --- | --- | --- | --- | --- | --- |
|  | **Unadjusted** | | | **Adjusted** | | |
|  | **OR (95% CI)** | **P-value** | **% Mediated*** | **OR (95% CI)** | **P-value** | **% Mediated*** |
| Total effect #1** | 1.34 (0.85 – 1.84) | 0.114 |  | 1.29 (0.81 – 1.78) | 0.175 |  |
| Total effect #2** | 1.81 (1.12 – 2.51) | 0.002 |  | 1.77 (1.07 – 2.46) | 0.004 |  |
| Direct effect #1 | 1.36 (0.86 – 1.86) | 0.099 |  | 1.31 (0.83 – 1.80) | 0.147 |  |
| Direct effect #2 | 1.88 (1.16 – 2.59) | 0.001 |  | 1.84 (1.12 – 2.56) | 0.002 |  |
| Indirect effect via CRP #1 | 1.00 (0.99 – 1.01) | 0.839 | NA | 1.00 (0.99 – 1.01) | 0.704 | NA |
| Indirect effect via CRP #2 | 1.00 (0.98 – 1.02) | 0.838 | NA | 1.00 (0.97 – 1.02) | 0.697 | NA |
| Indirect effect via IL-6 #1 | 1.00 (0.99 – 1.01) | 0.553 | NA | 1.00 (0.98 – 1.01) | 0.526 | NA |
| Indirect effect via IL-6 #2 | 1.00 (0.99 – 1.01) | 0.592 | 0.46 | 1.00 (0.99 – 1.02) | 0.540 | 0.65 |
| Indirect effect via suPAR #1 | 0.99 (0.96 – 1.02) | 0.623 | NA | 0.99 (0.96 – 1.02) | 0.566 | NA |
| Indirect effect via suPAR #2 | 0.97 (0.93 – 1.01) | 0.097 | NA | 0.96 (0.92 – 1.00) | 0.076 | NA |

Note: CI=confidence interval, CRP=C-reactive protein, IL-6=interleukin-6, OR=odds ratio, suPAR=soluble urokinase plasminogen activator receptor, *mediation percentages are marked NA (not applicable) where there is inconsistent mediation, i.e., the indirect effect OR is ≤1.00, leading to a negative percentage, ** #1 is the associations for the middle versus bottom level of urbanicity. #2 is the associations for the top versus bottom level of urbanicity. All analyses were conducted following multiple imputation and control for the non-independence of twin observations. Model adjustments are described in the Methods and in Figure 2.

**Supplementary Table 19. Association between air pollution and adolescent psychotic experiences, mediated via inflammation**

| **Inflammatory marker mediator** | **Associations split into total, direct and indirect effects and mediation percentage** | | | | | |
| --- | --- | --- | --- | --- | --- | --- |
|  | **Unadjusted** | | | **Adjusted** | | |
|  | **OR (95% CI)** | **P-value** | **% Mediated*** | **OR (95% CI)** | **P-value** | **% Mediated*** |
| Total effect #1** | 1.02 (0.69 – 1.36) | 0.887 |  | 1.04 (0.70 – 1.38) | 0.833 |  |
| Total effect #2** | 1.72 (1.20 – 2.24) | <0.001 |  | 1.69 (1.17 – 2.22) | 0.001 |  |
| Direct effect #1 | 1.01 (0.68 – 1.34) | 0.943 |  | 1.02 (0.69 – 1.36) | 0.884 |  |
| Direct effect #2 | 1.72 (1.20 – 2.24) | <0.001 |  | 1.71 (1.17 – 2.24) | 0.001 |  |
| Indirect effect via CRP #1 | 1.00 (0.98 – 1.01) | 0.779 | NA | 1.00 (0.98 – 1.01) | 0.634 | NA |
| Indirect effect via CRP #2 | 1.00 (0.97 – 1.02) | 0.778 | NA | 0.99 (0.97 – 1.02) | 0.626 | NA |
| Indirect effect via IL-6 #1 | 1.00 (0.99 – 1.01) | 0.878 | 2.45 | 1.00 (0.99 – 1.01) | 0.842 | 2.71 |
| Indirect effect via IL-6 #2 | 1.00 (0.99 – 1.01) | 0.510 | 0.65 | 1.00 (0.99 – 1.02) | 0.448 | 0.89 |
| Indirect effect via suPAR #1 | 1.01 (0.99 – 1.04) | 0.290 | 57.36 | 1.01 (0.99 – 1.04) | 0.313 | 40.51 |
| Indirect effect via suPAR #2 | 1.00 (0.97 – 1.02) | 0.768 | NA | 1.00 (0.97 – 1.02) | 0.717 | NA |

Note: CI=confidence interval, CRP=C-reactive protein, IL-6=interleukin-6, OR=odds ratio, suPAR=soluble urokinase plasminogen activator receptor, *mediation percentages are marked NA (not applicable) where there is inconsistent mediation, i.e., the indirect effect OR is ≤1.00, leading to a negative percentage, ** #1 is the associations for the middle versus bottom level of air pollution. #2 is the associations for the top versus bottom level of air pollution. All analyses were conducted following multiple imputation and control for the non-independence of twin observations. Model adjustments are described in the Methods and in Figure 2.

**Supplementary Table 20. Association between neighborhood deprivation and adolescent psychotic experiences, mediated via inflammation**

| **Inflammatory marker mediator** | **Associations split into total, direct and indirect effects and mediation percentage** | | | | | |
| --- | --- | --- | --- | --- | --- | --- |
|  | **Unadjusted** | | | **Adjusted** | | |
|  | **OR (95% CI)** | **P-value** | **% Mediated*** | **OR (95% CI)** | **P-value** | **% Mediated*** |
| Total effect #1** | 1.47 (1.02 – 1.93) | 0.014 |  | 1.43 (0.99 – 1.88) | 0.023 |  |
| Total effect #2** | 2.09 (1.38 – 2.80) | <0.001 |  | 1.97 (1.28 – 2.66) | <0.001 |  |
| Direct effect #1 | 1.45 (1.00 – 1.90) | 0.019 |  | 1.41 (0.97 – 1.85) | 0.031 |  |
| Direct effect #2 | 2.05 (1.36 – 2.75) | <0.001 |  | 1.94 (1.26 – 2.62) | <0.001 |  |
| Indirect effect via CRP #1 | 1.00 (1.00 – 1.00) | 0.900 | NA | 1.00 (0.99 – 1.00) | 0.837 | NA |
| Indirect effect via CRP #2 | 1.00 (0.99 – 1.01) | 0.774 | NA | 1.00 (0.99 – 1.01) | 0.681 | NA |
| Indirect effect via IL-6 #1 | 1.00 (0.99 – 1.01) | 0.594 | NA | 1.00 (0.99 – 1.01) | 0.612 | NA |
| Indirect effect via IL-6 #2 | 1.00 (0.98 – 1.01) | 0.604 | NA | 1.00 (0.98 – 1.01) | 0.691 | NA |
| Indirect effect via suPAR #1 | 1.02 (0.99 – 1.05) | 0.153 | 4.89 | 1.02 (0.99 – 1.05) | 0.159 | 5.42 |
| Indirect effect via suPAR #2 | 1.02 (0.99 – 1.06) | 0.172 | 3.08 | 1.02 (0.99 – 1.05) | 0.216 | 3.04 |

Note: CI=confidence interval, CRP=C-reactive protein, IL-6=interleukin-6, OR=odds ratio, suPAR=soluble urokinase plasminogen activator receptor, *mediation percentages are marked NA (not applicable) where there is inconsistent mediation, i.e., the indirect effect OR is ≤1.00, leading to a negative percentage, ** #1 is the associations for the middle versus bottom level of neighborhood deprivation. #2 is the associations for the top versus bottom level of neighborhood deprivation. All analyses were conducted following multiple imputation and control for the non-independence of twin observations. Model adjustments are described in the Methods and in Figure 2.

**Supplementary Table 21. Association between neighborhood disorder and adolescent psychotic experiences, mediated via inflammation**

| **Inflammatory marker mediator** | **Associations split into total, direct and indirect effects and mediation percentage** | | | | | |
| --- | --- | --- | --- | --- | --- | --- |
|  | **Unadjusted** | | | **Adjusted** | | |
|  | **OR (95% CI)** | **P-value** | **% Mediated*** | **OR (95% CI)** | **P-value** | **% Mediated*** |
| Total effect #1** | 1.28 (0.88 – 1.69) | 0.125 |  | 1.25 (0.85 – 1.65) | 0.172 |  |
| Total effect #2** | 1.73 (1.18 – 2.27) | 0.001 |  | 1.60 (1.09 – 2.11) | 0.004 |  |
| Direct effect #1 | 1.26 (0.86 – 1.66) | 0.151 |  | 1.23 (0.84 – 1.62) | 0.207 |  |
| Direct effect #2 | 1.67 (1.14 – 2.20) | 0.002 |  | 1.55 (1.05 – 2.05) | 0.008 |  |
| Indirect effect via CRP #1 | 1.00 (1.00 – 1.00) | 0.965 | 0.03 | 1.00 (0.99 – 1.01) | 0.979 | NA |
| Indirect effect via CRP #2 | 1.00 (1.00 – 1.01) | 0.789 | 0.14 | 1.00 (0.99 – 1.01) | 0.799 | 0.18 |
| Indirect effect via IL-6 #1 | 1.00 (0.99 – 1.02) | 0.489 | 1.85 | 1.01 (0.99 – 1.02) | 0.440 | 2.66 |
| Indirect effect via IL-6 #2 | 1.00 (0.99 – 1.01) | 0.739 | NA | 1.00 (0.99 – 1.01) | 0.842 | NA |
| Indirect effect via suPAR #1 | 1.01 (0.99 – 1.04) | 0.303 | 4.92 | 1.01 (0.99 – 1.04) | 0.342 | 5.43 |
| Indirect effect via suPAR #2 | 1.03 (0.99 – 1.08) | 0.105 | 6.11 | 1.04 (0.99 – 1.08) | 0.092 | 7.44 |

Note: CI=confidence interval, CRP=C-reactive protein, IL-6=interleukin-6, OR=odds ratio, suPAR=soluble urokinase plasminogen activator receptor, *mediation percentages are marked NA (not applicable) where there is inconsistent mediation, i.e., the indirect effect OR is ≤1.00, leading to a negative percentage, ** #1 is the associations for the middle versus bottom level of neighborhood disorder. #2 is the associations for the top versus bottom level of neighborhood disorder. All analyses were conducted following multiple imputation and control for the non-independence of twin observations. Model adjustments are described in the Methods and in Figure 2.

**Supplementary Table 22. Association between family disadvantage and adolescent psychotic experiences, mediated via inflammation**

| **Inflammatory marker mediator** | **Associations split into total, direct and indirect effects and mediation percentage** | | | | | |
| --- | --- | --- | --- | --- | --- | --- |
|  | **Unadjusted** | | | **Adjusted** | | |
|  | **OR (95% CI)** | **P-value** | **% Mediated*** | **OR (95% CI)** | **P-value** | **% Mediated*** |
| Total effect #1** | 1.80 (1.28 – 2.32) | <0.001 |  | 1.70 (1.20 – 2.20) | <0.001 |  |
| Total effect #2** | 2.17 (1.49 – 2.85) | <0.001 |  | 1.99 (1.32 – 2.67) | <0.001 |  |
| Direct effect #1 | 1.77 (1.25 – 2.28) | <0.001 |  | 1.66 (1.17 – 2.16) | 0.001 |  |
| Direct effect #2 | 2.14 (1.46 – 2.83) | <0.001 |  | 1.96 (1.28 – 2.64) | <0.001 |  |
| Indirect effect via CRP #1 | 1.00 (0.99 – 1.00) | 0.765 | NA | 1.00 (0.99 – 1.01) | 0.696 | NA |
| Indirect effect via CRP #2 | 1.00 (1.00 – 1.01) | 0.851 | 0.06 | 1.00 (0.99 – 1.01) | 0.998 | NA |
| Indirect effect via IL-6 #1 | 0.99 (0.98 – 1.01) | 0.434 | NA | 0.99 (0.98 – 1.01) | 0.403 | NA |
| Indirect effect via IL-6 #2 | 0.98 (0.95 – 1.01) | 0.270 | NA | 0.98 (0.94 – 1.02) | 0.238 | NA |
| Indirect effect via suPAR #1 | 1.03 (0.99 – 1.07) | 0.200 | 4.34 | 1.03 (0.99 – 1.07) | 0.135 | 5.80 |
| Indirect effect via suPAR #2 | 1.03 (0.98 – 1.08) | 0.189 | 4.14 | 1.04 (0.99 – 1.09) | 0.132 | 5.64 |

Note: CI=confidence interval, CRP=C-reactive protein, IL-6=interleukin-6, OR=odds ratio, suPAR=soluble urokinase plasminogen activator receptor, *mediation percentages are marked NA (not applicable) where there is inconsistent mediation, i.e., the indirect effect OR is ≤1.00, leading to a negative percentage, ** #1 is the associations for the middle versus bottom level of family disadvantage. #2 is the associations for the top versus bottom level of family disadvantage. All analyses were conducted following multiple imputation and control for the non-independence of twin observations. Model adjustments are described in the Methods and in Figure 2.

**Supplementary Table 23.** **Comparison between e-values and covariate associations for the socioeconomic risk-crystalized ability-psychotic experiences mediation model**

| **E-values/Covariates** | **Association with socioeconomic risk** | | **Association with crystalized ability** | | **Association with psychotic experiences** | |
| --- | --- | --- | --- | --- | --- | --- |
|  | **OR / B (OR), ^a^ (Lower CI / 95% CI** ^b^) | **P-value** | **OR / B (OR), ^a^ (Lower CI / 95% CI** ^b^) | **P-value** | **OR (Lower CI / 95% CI** ^b^) | **P-value** |
|  |  |  |  |  |  |  |
| E-value: Total effect | 2.61 (1.90) | - | 2.61 (1.90) | - | 2.61 (1.90) | - |
| E-value: Direct effect | 2.34 (1.64) | - | 2.34 (1.64) | - | 2.34 (1.64) | - |
| E-value: Indirect effect | 1.40 (1.21) | - | 1.40 (1.21) | - | 1.40 (1.21) | - |
|  |  |  |  |  |  |  |
| Biological sex at birth | -0.03 (0.97), (-0.10 – 0.03 (0.90 – 1.03)) | 0.312 | -0.83 (0.44), (-1.15 – -0.51 (0.32 – 0.60)) | <0.001 | 0.91 (0.73 – 1.13) | 0.382 |
| Family psychiatric history | 0.21 (1.24), (0.09 – 0.34 (1.09 – 1.40)) | 0.001 | -0.16 (0.85), (-0.75 – 0.42 (0.47 – 1.53)) | 0.585 | 2.44 (1.63 – 3.65) | <0.001 |
| Parental education | 0.55 (1.73), (0.46 – 0.64 (1.58 – 1.90)) | <0.001 | -1.76 (0.17), (-2.19 – -1.33 (0.11 – 0.26)) | <0.001 | 1.22 (0.92 – 1.62) | 0.164 |
| PRS for schizophrenia | -0.00 (1.00), (-0.03 – 0.03 (0.97 – 1.03)) | 0.979 | -0.09 (0.92), (-0.26 – 0.09 (0.77 – 1.09)) | 0.321 | 1.09 (0.98 – 1.22) | 0.124 |
| PRS for educational attainment | -0.09 (0.91), (-0.14 – -0.04 (0.87 – 0.96)) | <0.001 | 0.72 (2.05), (0.49 – 0.95 (1.63 – 2.58)) | <0.001 | 0.92 (0.78 – 1.08) | 0.294 |
| PRS for cognitive performance | -0.05 (0.95), (-0.10 – -0.00 (0.90 – 1.00)) | 0.037 | 0.51 (1.67), (0.28 – 0.74 (1.33 – 2.10)) | <0.001 | 0.88 (0.74 – 1.04) | 0.144 |

Note: ** p<0.01; *** p<0.001, ^a^ beta coefficients are presented alongside exponentiated coefficients (ORs) to allow comparison with E-values and associations for psychotic experiences, ^b^ E-values do not include p-values, but robustness is inferred from the lower CI (>1.00), B=beta, CI=confidence interval, OR=odds ratio, PRS=polygenic risk score.

**Supplementary Table 24.** **Complete case analysis: Association of socioenvironmental adversity with cognitive ability and inflammation**

| **Outcome/mediator** | **Association of physical risk with cognitive ability and inflammation** | | | | **Association of socioeconomic risk with cognitive ability and inflammation** | | | |
| --- | --- | --- | --- | --- | --- | --- | --- | --- |
|  | **Unadjusted** | | **Adjusted** | | **Unadjusted** | | **Adjusted** | |
|  | **B**  **(95% CI)** | **P-value** | **B**  **(95% CI)** | **P-value** | **B**  **(95% CI)** | **P-value** | **B**  **(95% CI)** | **P-value** |
| Overall cognitive ability | -1.14  (-2.53 – 0.26) | 0.110 | -0.63  (-1.86 – 0.59) | 0.311 | -8.72  (-10.05 – -7.39) | <0.001 | -5.74  (-7.05 – -4.43) | <0.001 |
| Crystalized ability | -0.37  (-0.68 – -0.07) | 0.016 | -0.27  (-0.53 – 0.00) | 0.053 | -1.99  (-2.28 – -1.71) | <0.001 | -1.39  (-1.69 – -1.10) | <0.001 |
| Fluid ability | -0.21  (-0.47 – 0.05) | 0.108 | -0.13  (-0.37 – 0.11) | 0.278 | -1.18  (-1.43 - -0.94) | <0.001 | -0.78  (-1.03 – -0.52) | <0.001 |
| Working memory | 0.02  (-0.28 – 0.32) | 0.880 | 0.08  (-0.20 – 0.37) | 0.565 | -1.13  (-1.43 - -0.84) | <0.001 | -0.67  (-0.97 – -0.37) | <0.001 |
| CRP | -0.12  (-0.27 – 0.03) | 0.129 | -0.12  (-0.27 – 0.04) | 0.132 | -0.00  (-0.16 – 0.15) | .965 | -0.01  (-0.17 – 0.16) | .924 |
| IL-6 | -0.04  (-0.11 – 0.02) | 0.178 | -0.04  (-0.11 – 0.02) | 0.162 | 0.09  (0.03 – 0.15) | 0.006 | 0.09  (0.02 – 0.16) | 0.009 |
| suPAR | -0.08  (-0.18 – 0.02) | 0.122 | -0.08  (-0.18 – 0.02) | 0.114 | 0.24  (0.14 – 0.35) | <0.001 | 0.23  (0.12 – 0.35) | <0.001 |

Note: B=beta, CI=confidence interval, CRP=C-reactive protein, IL-6=interleukin-6, suPAR=soluble urokinase plasminogen activator receptor. All analyses were conducted on those with complete data and control for the non-independence of twin observations. Complete case sample is N=1,794-1,797 for cognitive abilities and N=1,216-1,230 for inflammatory markers. Model adjustments are described in the Methods and in Figure 2.

.

**Supplementary Table 25.** **Complete case analysis: Association of cognitive ability and inflammation with adolescent psychotic experiences**

| **Cognitive ability/inflammatory marker** | **Association with psychotic experiences** | | | |
| --- | --- | --- | --- | --- |
|  | **Unadjusted** | | **Adjusted** | |
|  | **OR (95% CI)** | **P-value** | **OR (95% CI)** | **P-value** |
| Overall cognitive ability | 0.98 (0.98 – 0.99) | <0.001 | 0.99 (0.98 – 1.00) | 0.005 |
| Crystalized ability | 0.91 (0.88 – 0.94) | <0.001 | 0.93 (0.89 – 0.97) | <0.001 |
| Fluid ability | 0.96 (0.93 – 1.00) | 0.075 | 0.99 (0.95 – 1.03) | 0.588 |
| Working memory | 0.95 (0.92 – 0.98) | 0.003 | 0.96 (0.93 – 1.00) | 0.050 |
| CRP | 1.01 (0.92 – 1.11) | 0.858 | 1.01 (0.92 – 1.11) | 0.848 |
| IL-6 | 0.99 (0.81 – 1.20) | 0.903 | 0.97 (0.80 – 1.19) | 0.776 |
| suPAR | 1.14 (0.99 – 1.32) | 0.070 | 1.15 (0.99 – 1.34) | 0.066 |

Note: CI=confidence interval, CRP=C-reactive protein, IL-6=interleukin-6, OR=odds ratio, suPAR=soluble urokinase plasminogen activator receptor. All analyses were conducted on those with complete data and control for the non-independence of twin observations. Complete case sample is N=1,767-1,768 for cognitive abilities and N=1,257-1,271 for inflammatory markers. Model adjustments are described in the Methods and in Figure 2.

**Supplementary Table 26.** **Complete case analysis: Association between socioenvironmental adversity and adolescent psychotic experiences, mediated via cognitive ability and inflammation**

| **Socioenvironmental adversity factor score** | **Total, direct and indirect effects on adolescent psychotic experiences** | | | | | |
| --- | --- | --- | --- | --- | --- | --- |
| **Mediator** | **Unadjusted** | | | **Adjusted** | | |
|  | **OR (95% CI)** | **P-value** | **% Mediated*** | **OR (95% CI)** | **P-value** | **% Mediated*** |
| **Physical risk** |  |  |  |  |  |  |
| Overall cognitive ability |  |  |  |  |  |  |
| Total effect | 1.27 (1.02 – 1.51) | 0.018 |  | 1.24 (0.99 – 1.48) | 0.035 |  |
| Direct effect | 1.24 (1.00 – 1.48) | 0.029 |  | 1.23 (0.99 – 1.47) | 0.042 |  |
| Indirect effect via overall cognitive ability | 1.02 (0.99 – 1.04) | 0.134 | 7.75 | 1.01 (0.99 – 1.02) | 0.335 | 3.52 |
| Specific cognitive abilities |  |  |  |  |  |  |
| Total effect | 1.27 (1.02 – 1.52) | 0.018 |  | 1.24 (0.99 – 1.49) | 0.034 |  |
| Direct effect | 1.24 (0.99 – 1.48) | 0.037 |  | 1.22 (0.98 – 1.47) | 0.049 |  |
| Indirect effect via crystalized ability | 1.03 (1.00 – 1.07) | 0.046 | 13.55 | 1.02 (1.00 – 1.04) | 0.116 | 8.87 |
| Indirect effect via fluid ability | 1.00 (0.98 – 1.01) | 0.401 | NA | 1.00 (0.99 – 1.01) | 0.432 | NA |
| Indirect effect via working memory | 1.00 (0.99 – 1.01) | 0.880 | NA | 1.00 (0.99 – 1.00) | 0.622 | NA |
| Inflammatory markers |  |  |  |  |  |  |
| Total effect | 1.41 (1.08 – 1.73) | 0.004 |  | 1.38 (1.05 – 1.70) | 0.008 |  |
| Direct effect | 1.42 (1.10 – 1.75) | 0.002 |  | 1.40 (1.07 – 1.72) | 0.005 |  |
| Indirect effect via CRP | 1.00 (0.98 – 1.01) | 0.686 | NA | 1.00 (0.98 – 1.01) | 0.551 | NA |
| Indirect effect via IL-6 | 1.01 (0.99 – 1.02) | 0.343 | 1.76 | 1.01 (0.99 – 1.02) | 0.277 | 2.42 |
| Indirect effect via suPAR | 0.99 (0.96 – 1.01) | 0.195 | NA | 0.98 (0.96 – 1.01) | 0.187 | NA |
| **Socioeconomic risk** |  |  |  |  |  |  |
| Overall cognitive ability |  |  |  |  |  |  |
| Total effect | 1.81 (1.46 – 2.15) | <0.001 |  | 1.65 (1.30 – 2.00) | <0.001 |  |
| Direct effect | 1.67 (1.34 – 2.00) | <0.001 |  | 1.57 (1.23 – 1.91) | <0.001 |  |
| Indirect effect via overall cognitive ability | 1.08 (1.00 – 1.16) | 0.034 | 13.48 | 1.05 (0.99 – 1.10) | 0.076 | 9.19 |
| Specific cognitive abilities |  |  |  |  |  |  |
| Total effect | 1.81 (1.46 – 2.15) | <0.001 |  | 1.64 (1.29 – 2.00) | <0.001 |  |
| Direct effect | 1.63 (1.30 – 1.96) | <0.001 |  | 1.54 (1.20 – 1.88) | <0.001 |  |
| Indirect effect via crystalized ability | 1.13 (1.03 – 1.23) | 0.009 | 20.85 | 1.08 (1.01 – 1.16) | 0.022 | 16.04 |
| Indirect effect via fluid ability | 0.96 (0.91 – 1.02) | 0.174 | NA | 0.98 (0.94 – 1.01) | 0.194 | NA |
| Indirect effect via working memory | 1.02 (0.97 – 1.07) | 0.443 | 3.14 | 1.01 (0.98 – 1.04) | 0.476 | 2.13 |
| Inflammatory markers |  |  |  |  |  |  |
| Total effect | 1.67 (1.30 – 2.05) | <0.001 |  | 1.61 (1.22 – 2.01) | <0.001 |  |
| Direct effect | 1.65 (1.27 – 2.03) | <0.001 |  | 1.59 (1.19 – 1.98) | <0.001 |  |
| Indirect effect via CRP | 1.00 (0.99 – 1.00) | 0.948 | NA | 1.00 (0.99 – 1.01) | 0.924 | NA |
| Indirect effect via IL-6 | 0.98 (0.96 – 1.01) | 0.188 | NA | 0.98 (0.95 – 1.01) | 0.164 | NA |
| Indirect effect via suPAR | 1.03 (0.99 – 1.07) | 0.116 | 6.17 | 1.04 (0.99 – 1.08) | 0.099 | 7.13 |

Note: CI=confidence interval, CRP=C-reactive protein, IL-6=interleukin-6, OR=odds ratio, suPAR=soluble urokinase plasminogen activator receptor, *mediation percentages are marked NA (not applicable) where there is inconsistent mediation, i.e., the indirect effect OR is ≤1.00, leading to a negative percentage. All analyses were conducted on those with complete data and control for the non-independence of twin observations. Complete case sample is N=1,699 for cognitive abilities and N=1,199 for inflammatory markers. Model adjustments are described in the Methods and in Figure 2. For mediation models, overall cognitive ability was included as a separate mediator. In contrast, specific cognitive abilities were included simultaneously to account for potentially overlapping pathways. Likewise, inflammatory markers were included simultaneously. Note that total effects differ slightly across models due to differences in model specifications and sample sizes.

**References from Supplementary Materials**

**1.** Trouton A, Spinath FM, Plomin R. Twins early development study (TEDS): A multivariate, longitudinal genetic investigation of language, cognition and behavior problems in childhood. *Twin Res.* 2002;5:444-448.

**2.** Maynard RA. *Kids having kids: Economic costs & social consequences of teen pregnancy*. Washington, DC: The Urban Insitute; 1997.

**3.** Moffitt TE, The E-Risk Study Team. Teen‐aged mothers in contemporary Britain. *J Child Psychol Psychiatry.* 2002;43:727-742.

**4.** Bennett N, Jarvis L, Rowlands O, Singleton N, Haselden L. *Living in Britain: Results from the 1994 general household survey*. London: HMSO; 1996.

**5.** Price TS, Freeman B, Craig I, Petrill SA, Ebersole L, Plomin R. Infant zygosity can be assigned by parental report questionnaire data. *Twin Res Hum Genet.* 2000;3:129-133.

**6.** Office for National Statistics. Urban and rural area definitions for policy purposes in England and Wales: Methodology (v1.0). https://assets.publishing.service.gov.uk/government/uploads/system/uploads/attachment_data/file/239477/RUC11methodologypaperaug_28_Aug.pdf; 2013.

**7.** Beevers SD, Kitwiroon N, Williams ML, Carslaw DC. One way coupling of CMAQ and a road source dispersion model for fine scale air pollution predictions. *Atmos Environ* 2012;59:47-58.

**8.** Carslaw DC. Defra urban model evaluation analysis - Phase 1. <https://uk-air.defra.gov.uk/library/reports?report_id=654>; 2011.

**9.** Newbury JB, Arseneault L, Beevers S, et al. Association of air pollution exposure with psychotic experiences during adolescence. *JAMA Psychiatry.* 2019;76:614-623.

**10.** Caspi A, Taylor A, Moffitt TE, Plomin R. Neighborhood deprivation affects children's mental health: Environmental risks identified in a genetic design. *Psychol Sci.* 2000;11:338-342.

**11.** Odgers CL, Moffitt TE, Tach LM, et al. The protective effects of neighborhood collective efficacy on British children growing up in deprivation: A developmental analysis. *Dev Psychol.* 2009;45:942-957.

**12.** Odgers CL, Caspi A, Russell MA, Sampson RJ, Arseneault L, Moffitt TE. Supportive parenting mediates neighborhood socioeconomic disparities in children's antisocial behavior from ages 5 to 12. *Dev Psychopathol.* 2012;24:705-721.

**13.** Jaffee SR, Caspi A, Moffitt TE, Polo-Tomas M, Taylor A. Individual, family, and neighborhood factors distinguish resilient from non-resilient maltreated children: A cumulative stressors model. *Child Abuse Negl.* 2007;31:231-253.

**14.** Sampson RJ, Raudenbush SW. Systematic social observation of public spaces: A new look at disorder in urban neighborhoods. *Am J Sociol.* 1999;105:603-651.

**15.** Poulton R, Caspi A, Moffitt TE, Cannon M, Murray R, Harrington H. Children's self-reported psychotic symptoms and adult schizophreniform disorder: A 15-year longitudinal study. *Arch Gen Psychiatry.* 2000;57:1053-1058.

**16.** Schreier A, Wolke D, Thomas K, et al. Prospective study of peer victimization in childhood and psychotic symptoms in a nonclinical population at age 12 years. *Arch Gen Psychiatry.* 2009;66:527-536.

**17.** Loewy RL, Pearson R, Vinogradov S, Bearden CE, Cannon TD. Psychosis risk screening with the Prodromal Questionnaire—brief version (PQ-B). *Schizophr Res.* 2011;129:42-46.

**18.** Miller TJ, McGlashan TH, Rosen JL, et al. Prodromal assessment with the structured interview for prodromal syndromes and the scale of prodromal symptoms: predictive validity, interrater reliability, and training to reliability. *Schizophr Bull.* 2003;29:703-715.

**19.** Miller T. The SIPS-Screen: a brief self-report screen to detect the schizophrenia prodrome. *Schizophr Res.* 2004:78.

**20.** Spauwen J, Krabbendam L, Lieb R, Wittchen HU, van Os J. Does urbanicity shift the population expression of psychosis? *J Psychiatr Res.* 2004;38:613-618.

**21.** Yoshizumi T, Murase S, Honjo S, Kaneko H, Murakami T. Hallucinatory experiences in a community sample of Japanese children. *J Am Acad Child Adolesc Psychiatry.* 2004;43:1030-1036.

**22.** Yung AR, Nelson B, Baker K, Buckby JA, Baksheev G, Cosgrave EM. Psychotic-like experiences in a community sample of adolescents: Implications for the continuum model of psychosis and prediction of schizophrenia. *Aust N Z J Psychiatry.* 2009;43:118-128.

**23.** Rasmussen LJH, Petersen JEV, Eugen-Olsen J. Soluble urokinase plasminogen activator receptor (suPAR) as a biomarker of systemic chronic inflammation. *Front Immunol.* 2021;12:780641.

**24.** Weissman MM, Wickramaratne P, Adams P, Wolk S, Verdeli H, Olfson M. Brief screening for family psychiatric history: The family history screen. *Arch Gen Psychiatry.* 2000;57:675-682.

**25.** Milne B, Moffitt T, Crump R, et al. How should we construct psychiatric family history scores? A comparison of alternative approaches from the Dunedin Family Health History Study. *Psychol Med.* 2008;38:1793-1802.

**26.** Howie BN, Donnelly P, Marchini J. A flexible and accurate genotype imputation method for the next generation of genome-wide association studies. *PLoS Genet.* 2009;5:e1000529.

**27.** Sherry ST, Ward M-H, Kholodov M, et al. dbSNP: the NCBI database of genetic variation. *Nucleic Acids Res.* 2001;29:308-311.

**28.** Dudbridge F. Power and predictive accuracy of polygenic risk scores. *PLoS Genet.* 2013;9:e1003348.

**29.** Euesden J, Lewis CM, O’Reilly PF. PRSice: polygenic risk score software. *Bioinformatics.* 2015;31:1466-1468.

**30.** Pardiñas AF, Holmans P, Pocklington AJ, et al. Common schizophrenia alleles are enriched in mutation-intolerant genes and in regions under strong background selection. *Nat Genet.* 2018;50:381-389.

**31.** Chang CC, Chow CC, Tellier LC, Vattikuti S, Purcell SM, Lee JJ. Second-generation PLINK: rising to the challenge of larger and richer datasets. *Gigascience.* 2015;4:s13742-13015-10047-13748.

**32.** Becker J, Burik CA, Goldman G, et al. Resource profile and user guide of the Polygenic Index Repository. *Nat Human Behav.* 2021:1-15.

**33.** Koyanagi A, Stickley A. The association between sleep problems and psychotic symptoms in the general population: a global perspective. *Sleep.* 2015;38:1875-1885.

**34.** Lee JJ, Wedow R, Okbay A, et al. Gene discovery and polygenic prediction from a genome-wide association study of educational attainment in 1.1 million individuals. *Nat Genet.* 2018;50:1112-1121.

**35.** Rietveld CA, Medland SE, Derringer J, et al. GWAS of 126,559 individuals identifies genetic variants associated with educational attainment. *Science.* 2013;340:1467-1471.

**36.** Trampush JW, Yang MLZ, Yu J, et al. GWAS meta-analysis reveals novel loci and genetic correlates for general cognitive function: a report from the COGENT consortium. *Mol Psychiatry.* 2017;22:336-345.

**37.** Savage JE, Jansen PR, Stringer S, et al. Genome-wide association meta-analysis in 269,867 individuals identifies new genetic and functional links to intelligence. *Nat Genet.* 2018;50:912-919.

**38.** Turley P, Walters RK, Maghzian O, et al. Multi-trait analysis of genome-wide association summary statistics using MTAG. *Nat Genet.* 2018;50:229-237.
